# Structural Prediction of Coronavirus s2m Kissing Complexes and Extended Duplexes

**DOI:** 10.1021/acsphyschemau.5c00031

**Published:** 2025-06-05

**Authors:** Adam H. Kensinger, Joseph A. Makowski, Mihaela Rita Mihailescu, Jeffrey D. Evanseck

**Affiliations:** Department of Chemistry and Biochemistry, 6613 Duquesne University, Pittsburgh, Pennsylvania 15282, United States

**Keywords:** RNA modeling, hybrid structure prediction, atomistic ensemble generation, COVID-19, HIV

## Abstract

The three-dimensional (3D) atomistic-resolution structure and dynamics of RNA kissing complexes (KCs) and extended duplexes (EDs), homodimers formed through palindromic base pairing, are crucial for understanding viral replication and structure-informed therapeutic design. Polyacrylamide gel electrophoresis (PAGE) evidence suggests KC and ED dimer formation between stem-loop II motif (s2m) elements in SARS-CoV, SARS-CoV-2, and Delta SARS-CoV-2, which may regulate host immune response. However, the absence of 3D structural data on s2m dimers limits structural interpretation needed to explain differences in stability indicated by native PAGE and biophysical implications. In this work, we evaluate the VFold3D/LA-IsRNA pipeline for resolving 3D structures of s2m KCs and EDs by validating its accuracy with blind and referenced predictions against experimental HIV-1 DIS KC and ED structures. Engendering confidence in the approach for blind prediction of KC and ED structures, HIV-1 DIS predictions achieved an average RMSD of 3.28 Å relative to crystal structures, while local interactions, such as palindrome-flanking purine stack orientations in the terminal loops, were in closer agreement with reported solution-phase NMR (RMSD ∼ 2.5 Å), cryo-EM maps, and previous molecular dynamics (MD) simulations. We find that the predicted 3D dimer structures of s2m resulted in kinked or linear shapes of s2m KC complexes that provide an interpretation consistent with native PAGE migration differences, where KCs are more kinked (63° to 133°) than linear ED dimers (127° to 156°). Following MD refinement, the SARS-CoV s2m KC adopts stacking palindromic basepair triplets, whereas SARS-CoV-2 and Delta s2m only form canonical palindrome basepairs, explaining their relative dimer instability suggested by PAGE band intensity. Ultimately, our results support the use of the VFold3D/LA-IsRNA pipeline for KC and ED generation, yielding predictions consistent with experimental data and providing an atomistic foundation for data-driven design of antiviral therapies to disrupt the lifecycle or immune response of viruses.

## Graphical abstract

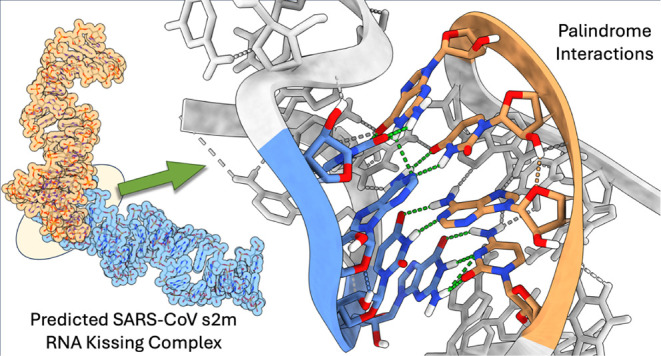

## 1 Introduction

RNA kissing complexes (KC) and extended duplexes (ED) are dynamic, interconverting dimers formed through base pairing of complementary hairpin loops (Figure 1).^1^ Specifically, the formation of KC structures is often facilitated through a palindromic sequence in the hairpin terminal loop, which form canonical base pairs when inverted – as is the case when separate hairpin monomers come into contact through space. The corresponding ED structure forms analogous base pairs through duplex topology rather than between or within distinct hairpins. These structures play essential roles in the life cycles of various viruses and have emerged as potential therapeutic targets.^2–4^ However, the complexity of heterogeneous RNA energy landscapes^4^ has hindered structural characterization of many biologically relevant dimers, including those involving the stem-loop II motif (s2m) found in the 3′ untranslated regions of SARS-CoV, SARS-CoV-2, and the SARS-CoV-2 Delta variant.^5–10^ Despite experimental evidence that the s2m forms dimers, proposed to be KCs and EDs, via its palindromic GUAC terminal loop sequence,^4–9^ no high-resolution three-dimensional structural data has been reported for the s2m in its dimeric states. Given the demonstrated ability of s2m to bind host microRNAs, suggesting a role in modulating the host immune response,^11,12^ understanding the structure of s2m dimers is critical to elucidating the biophysical underpinnings of its role in the viral lifecycle or immune response.

**Figure 1:**
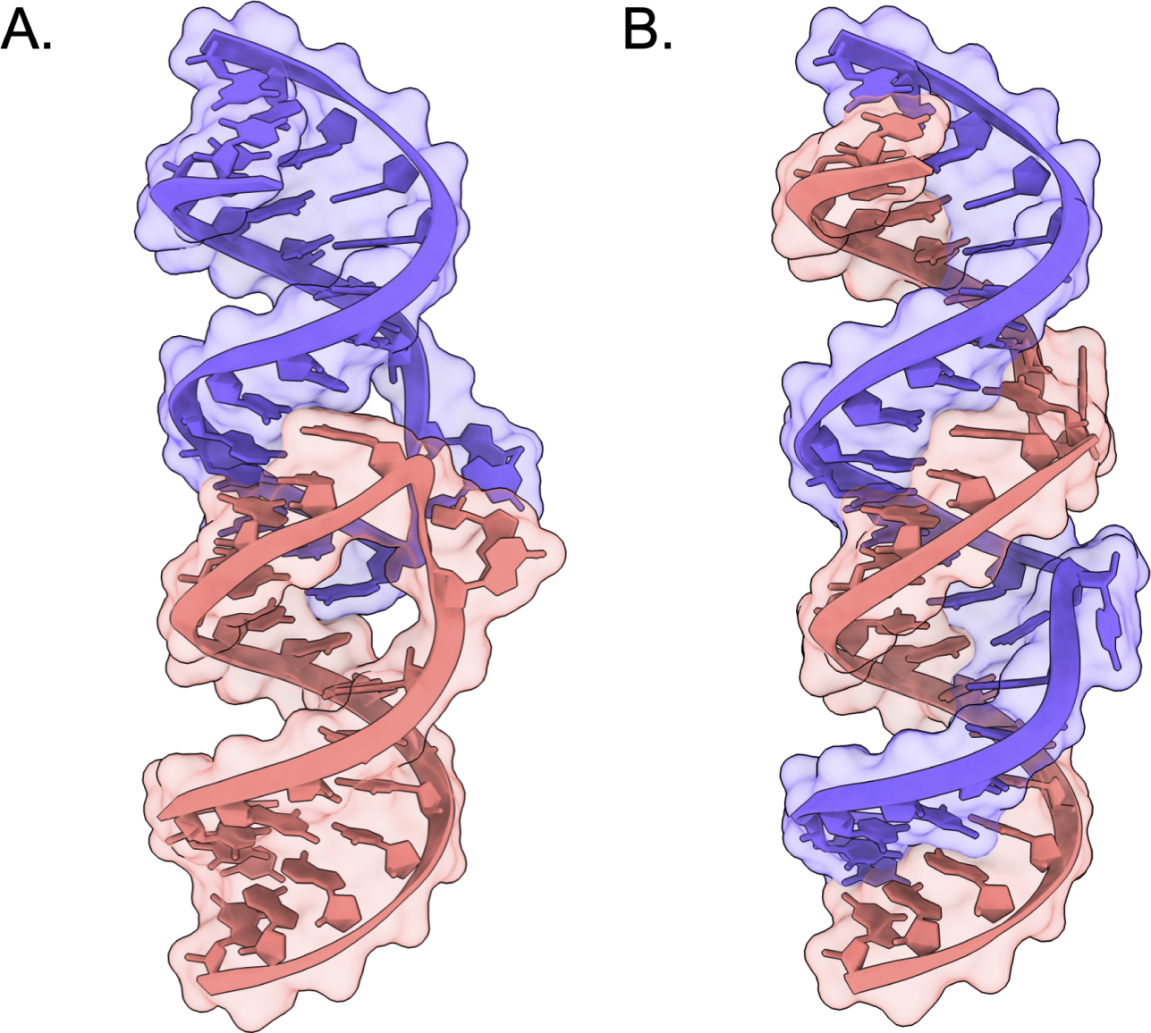
Representative KC and ED structures (PDBs 1XPF and 1Y99, respectively).

Determining accurate starting atomic coordinates are necessary for generation of structural ensembles to characterize the structure, dynamics, and energetics of dimers using molecular dynamics MD simulation trajectories, which are highly sensitive to initial conditions.^13^ However, a major obstacle to modeling such systems lies in the limitations of existing RNA structure prediction tools.^14^ Most currently available software implementations of RNA 3D structure prediction remain limited to short RNA sequences, single stranded RNAs, or simple duplex RNAs that do not include higher order tertiary structures like pseudoknots or kissing interactions.^14^ Even those available through webservers can be impractical due to long prediction times.^14^ While recent progress in deep learning has transformed protein structure prediction,^15^ these data-intensive methods remain hampered for RNA applications by the scarcity of RNA structures in the Protein Data Bank (PDB) for training purposes.^15^ As a consequence, many deep learning implementations of RNA prediction software are limited to either single-stranded RNAs or topologically simple duplex structures, excluding the possibility for prediction of biologically important structures that include pseudoknot or kissing interactions.^16^ Additionally, purely physics-based RNA folding is intractable due to the computational complexity of fully atomistic simulations with many atoms.^17^ These limitations have driven interest in hybrid approaches, such as the VFold3D/LA-IsRNA pipeline, which combines template-based modeling and coarse-grained physics with efficient support for multistrand RNAs and practical runtimes.^18,19^

The dimerization initiation site (DIS) of human immunodeficiency virus 1 (HIV-1), with over 40 deposited KC and ED PDB structures,^3,4,20–28^ is routinely chosen as a structural standard for benchmarking RNA KC prediction methods.^29–32^ Initial benchmarking studies of the Vfold model demonstrated its ability to predict HIV-1 DIS structures (subtypes A, B, and F KCs and EDs) within ∼3.0 Å RMSD of experimental data, showing good agreement with thermal stability measurements from experimental melting temperatures.^31,32^ Recent hybrid methods, which integrate template-based Vfold3D/LA models with the physics-based iterative simulated reference state (IsRNA) model, have enabled accurate predictions of medium-sized RNAs (22–78 nts) with complex topologies.^18^ This combined approach goes beyond static predictions by leveraging coarse-grained MD simulations to generate ensemble geometries for atomistic simulations under realistic solvent and ionic conditions,^18,19^ known to be important in forming RNA kissing interactions.^12^ Building upon the previous benchmarking of Vfold against HIV-1 DIS, our intention in this study is to provide a contemporary assessment of the hybrid Vfold3D/LA-IsRNA pipeline specifically for the prediction of s2m KC and ED dimers.^31,32^

In this study, we assess the performance of the VFold3D/LA-IsRNA pipeline,^18,19,33^ for predicting the 3D structures of the KCs and EDs formed by the s2m element in SARS-CoV, SARS-CoV-2, and Delta SARS-CoV-2. As a benchmark, we evaluate prediction accuracy against known HIV-1 DIS KC and ED structures, using both blind and reference-based modeling modes.^34^ These comparisons validate the pipeline’s reliability in capturing key structural features of RNA hairpin dimers, building confidence in its applicability to systems lacking experimental structures. We extended this methodology to predict SARS-CoV, SARS-CoV-2, and the Delta variant s2m dimers, using the IsRNA empirical scoring function to assess structural predictions. The top three predicted s2m KCs from each system were used as inputs for microsecond-scale MD simulations to analyze solution-phase dynamics, which we found to be aligned with reported native PAGE data, rationalizing differences in complex migration. Ultimately, our results establish a predictive framework for exploring RNA dimer structure and dynamics through the VFold3D/LA-IsRNA pipeline and MD simulation, addressing the biophysical challenge of obtaining meaningful three-dimensional structural data for RNA hairpin complexes.

## 2 Methods

### 2.1 RNA 3D Structure Prediction and Validation

Although deep learning (DL)-based methods, such as AlphaFold,^35^ are anticipated to revolutionize RNA structure prediction, physics-based methods currently outperform DL models in RNA Critical Assessment of Structure Prediction (CASP) challenges.^36–38^ Unlike DL methods, which typically generate static structures, physics-based approaches provide conformational ensembles that better capture RNA’s dynamic nature.^18^ For our KC and ED predictions, we used the hybrid Vfold3D/LA-IsRNA pipeline, which leverages these strengths. The motif template-based Vfold3D model and the loop template-based VFoldLA model were developed specifically for noncoding RNA 3D prediction with secondary structure constraints, incorporating conserved 3D structural motifs.^32,33^ These initial predictions were further refined with the iterative simulated reference state (IsRNA) coarse-grained model MD simulation method, which generates a structural ensemble from a four/five-bead RNA model.^18,19^

The Vfold3D/LA-IsRNA pipeline, graphically summarized in Figure 2, allows for the submission of multiple sequences and expanded dot-bracket secondary structural data, with the flexibility to exclude PDBs from the template library to minimize bias from specific structures.^18,19^ To evaluate the pipeline, we performed both referenced assessment relative to known PDBs, and blind screening, which estimates plausibility when no experimental structures are available.^18^ Referenced assessment measures structural accuracy of a prediction to a known experimental structure, assessed by metrics such as RMSD of global and local folds; interaction network fidelity (INF), which quantifies the accuracy with which the predicted interactions between nucleotides match the experimentally observed interaction network; clash score, representing the steric component; and significance P-score, yielding a total empirical score.^39–41^ Blind screening measures how reliable a predicted structure is when no experimental structure is available, assessed by energy scoring functions, internal plausibility, ensemble convergence, and comparison to known homologous structures.^18^

**Figure 2:**
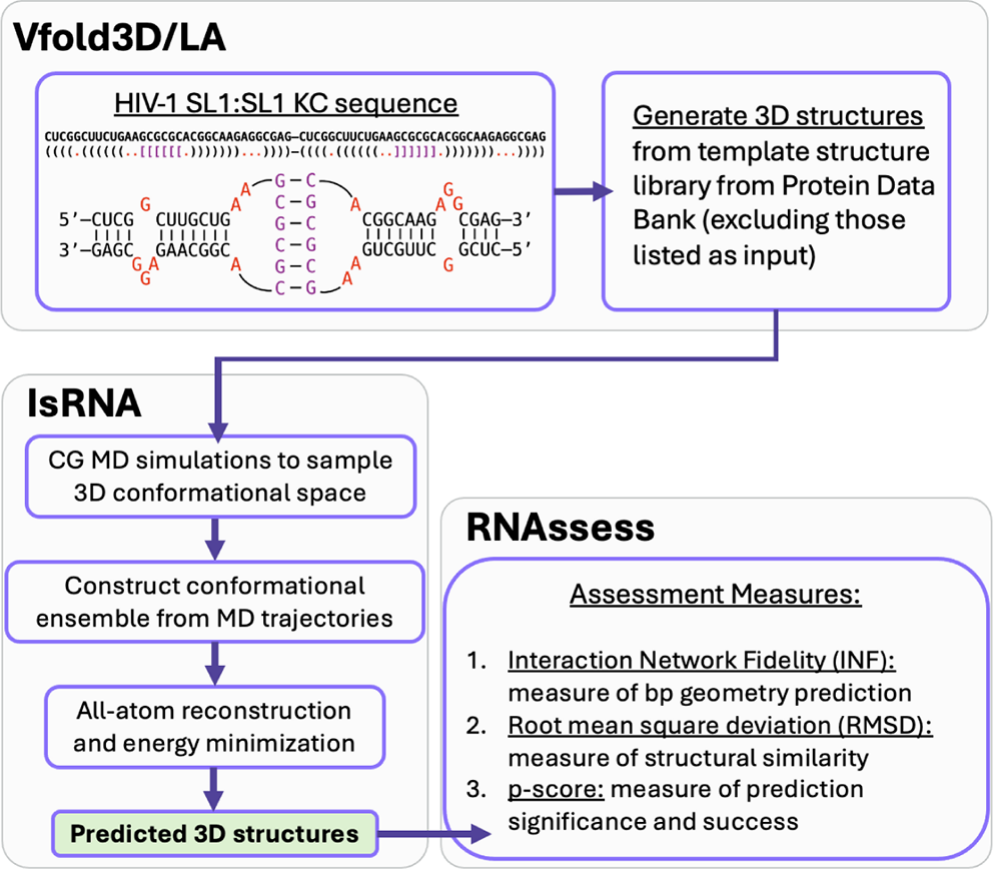
Vfold3D/LA-IsRNA pipeline.^33,42,43^ Predictions are initialized by the user providing a sequence and (extended) dot-bracket depiction of the RNA structure. Except for PDBs excluded by the user, an initial 3D structure is constructed using template fragments from the Protein Data Bank. Refinement into the final set of predicted structures is achieved using IsRNA with scoring metrics provided by RNAssess.

To test the reliability of the Vfold3D/LA-IsRNA pipeline to generate RNA dimer structures, the method was used to predict the DIS KC and ED structures of each HIV-1 subtype for which there is a PDB (including subtypes A, B, and F). To evaluate the influence of allowing those PDBs to be included as templates in the Vfold3D/LA modules, analogous predictions were made while excluding all 20 HIV-1 DIS KCs and EDs from the IsRNA structural template library. Validated predictions from HIV-1 subtypes would demonstrate that the Vfold3D/LA-IsRNA method generates trustworthy RNA dimer ensembles and that blind screening methods provide meaningful metrics of structural plausibility. This establishes a foundation for applying the pipeline to unresolved systems, such as the SARS-CoV, SARS-CoV-2, and Delta s2m structures.

### 2.2 Referenced Structure Prediction and Assessment Using HIV-1 DIS

The PDB contains structures from three subtypes (A, B, F) of the HIV-1 DIS,^44^ which each have unique monomer SL1 nucleotide sequences that were used for prediction input (Table 1). Subtype A and B are often referred to as Mal and Lai in the literature,^45^ respectively, but will be noted here with the letter nomenclature for clarity.

**Table 1:** HIV-1 DIS SL1 Monomer Sequences^a^

Subtype	SL1 sequence
A	CUUGCUGA **G** GUGCACACAGCAAG
B	CUUGCUGAAG **C** GC **G** CAC **G** GCAAG
F	CUUGCUGAAGUGCACACAGCAAG

a Some sequences of PDB structures include additional bases on the 3′ or 5′ ends beyond those listed here for various experimental reasons.

In total, the Protein Data Bank contains 25 HIV-1 KC structures and 21 HIV-1 ED structures from crystallography or solution-phase NMR experiments,^3,4,20–28^ but only 20 are included in the IsRNA template library due to redundancy (Table S1).^18,19^ For instance, groups of KC structures from a single study report nearly identical structures, but were cocrystallized with different salts or compounds.^3,4,20^ The library also contains structures for the HIV-1 transactivation response (TAR) element, which forms KC structures,^46–49^ and were included in the list of excluded PDB structure templates for falling under the category of HIV-1 KC.

For a given HIV-1 subtype, five predictions were made for both KC and ED structures either including the above list of PDBs as templates or excluding the list from the template library. Thus, the resulting bulk data included 30 KC predictions and 30 ED predictions. Default settings for the IsRNA Web server were used including 20 ns simulation time, 1000 snapshots recorded per replica, 10% of the lowest-energy snapshots were taken for clustering with an RMSD cutoff of 5 Å.^18^ No initial 3D structures were used as input for the simulations, and the input sequences and secondary structural dot-bracket notations are given in Table S2.

Final predictions were evaluated with a referenced assessment using the RNAssess Web server^34^ to compare directly back to experimental structures with criteria including RMSD, Interaction Network Fidelity (INF),^41^ clash score,^50^ and P-score.^40^ RMSD serves to measure the global structural similarity if calculated over all atoms, or locally as calculated through the local-neighborhood cutoff analysis. INF serves as a measure of the reproduction of local base-pair and stacking geometries of the nucleobases defined through the Leontis–Westhof notation and is represented by a value between zero and one,^51,52^ with values close to one indicating better agreement with the experimental base geometries.^41^ Clash score is calculated as the number of van der Waal radii overlaps per 1000 atoms.^50^ Finally, *P*-score can be calculated for RNA predictions with sequence length between 35 and 161 nucleotides and can evaluate prediction significance or success (*P* ≤ 0.01 indicates a significant prediction).^40^ Details regarding the calculation of these metrics are provided in the Supporting Information and reviewed in detail elsewhere.^34^ High resolution crystal structure PDB IDs for assessment comparison for each type of prediction are given in Table S2. Final predictions were further evaluated with the Structural Assessment module of the IsRNA Web server with default settings of 0.5 ns simulation time, 1000 snapshots recorded per trajectory, 5 duplicate simulations per model, and an energy threshold to filter good models of <0.975 × *E*_min_ for the energy scoring function.^18^

### 2.3 Referenced Structure Prediction and Assessment Extended to SARS-CoV, SARS-CoV-2, and Delta s2m

*De novo* structural predictions were made for the SARS-CoV, SARS-CoV-2, and Delta variant s2m elements with the same IsRNA input settings as for the HIV-1 DIS predictions above, with the exception that no PDBs were excluded from the template library. Importantly, the crystal structure of the SARS-CoV s2m monomer, 1XJR,^8^ is included in the IsRNA template library and was used for all predictions to improve accuracy. The respective 41-nucleotide sequences used were previously reported and the secondary structures have been proposed based on experimental native PAGE results revealing s2m dimerization properties and NMR secondary structures of the s2m monomers (Table 2).^5,12^

**Table 2:** IsRNA Input s2m KC or ED Sequences and Secondary Structures

Structure	Virus	s2m sequence/extended dot bracket
KC	SARS-CoV	UUCAUCGAGGCCACGCGGAGUACGAUCGAGGGUACAGUGAA-UUCAUCGAGGCCACGCGGAGUACGAUCGAGGGUACAGUGAA ((((((..((((((((···[[[[)..)).)))).)))))))-((((((..((((((((···]]]])..)).)))).)))))))
ED	SARS-CoV	UUCAUCGAGGCCACGCGGAGUACGAUCGAGGGUACAGUGAA-UUCAUCGAGGCCACGCGGAGUACGAUCGAGGGUACAGUGAA ((((((..((((((((···(((((..((.((((.(((((((−))))))..))))))))···)))))..)).)))).)))))))
KC	SARS-CoV-2	UUCACCGAGGCCACGCGGAGUACGAUCGAGUGUACAGUGAA-UUCACCGAGGCCACGCGGAGUACGAUCGAGUGUACAGUGAA (((((···..(((.((..[[[[···)).)))···)))))-(((((···..(((.((..]]]]···)).)))···)))))
ED	SARS-CoV-2	UUCACCGAGGCCACGCGGAGUACGAUCGAGUGUACAGUGAA-UUCACCGAGGCCACGCGGAGUACGAUCGAGUGUACAGUGAA (((((···..(((.((..((((···((.(((···(((((−)))))···..))).))..))))···)).)))···)))))
KC	Delta variant	UUCACCGAGGCCACUCGGAGUACGAUCGAGUGUACAGUGAA-UUCACCGAGGCCACUCGGAGUACGAUCGAGUGUACAGUGAA (((((···.((((((((.[[[[..))))))))···)))))-(((((···.((((((((.]]]]..))))))))···)))))
ED	Delta variant	UUCACCGAGGCCACUCGGAGUACGAUCGAGUGUACAGUGAA-UUCACCGAGGCCACUCGGAGUACGAUCGAGUGUACAGUGAA (((((···.((((((((((((((.((((((((···(((((−)))))···.)))))))))))))).))))))))···)))))

The ED for SARS-CoV s2m was not observed to form experimentally in the native PAGE, indicating that the KC is the only form of dimer, but in this study, the structure was predicted for comparison purposes.^12^

An approximate experimental structure comparison could be made for the SARS-CoV s2m monomers by calculating RMSD of each monomer in a predicted KC using the SARS-CoV s2m 1XJR crystal structure as reference 8. For this calculation, terminal loop nucleotides 17 to 27 were excluded to assess the similarity of structure in the stem regions only. The terminal loops are involved in the kissing interactions and are not expected to have the same structure as an individual monomeric s2m.

### 2.4 Molecular Dynamics Simulations

Each of the three lowest energy KC structures from the SARS-CoV, SARS-CoV-2, and Delta s2m predictions and screening were used as initial starting coordinates for unbiased 1 μs MD simulations. Our simulation protocol is analogous to our simulations of the s2m monomer systems, as described previously.^53,54^ The AMBER force field with the ff99 bsc0 χOL3 parameter set was employed through the NAMD molecular dynamics (MD) engine.^55–59^ To solvate each system, 15 Å of TIP3P water padding was used to solvate each system with periodic boundary conditions.^60^ The ionic atmosphere surrounding RNA structures in solution can impact structure, dynamics, and function and is important to consider carefully in MD simulation setup for meaningful interpretation.^61–63^ The formation of KC and ED structures has been shown to depend on concentration of Mg^2+^ ions, so the concentration chosen for the simulations in this study (4 Mg^2+^ ions, ca. 6.5 mM) was based on reported concentrations in native PAGE experiments including Mg^2+^ (1–10 mM) in which the structures were observed to form.^12^ Structural and dynamical dependence of KC formation on various levels of [Mg^2+^] is of interest in future studies, but was outside the scope of this work. Systems were charge neutralized and ionized to a concentration of approximately 150 mM NaCl, with Li/Merz parameters.^64^ Simulations were performed at physiological 310 K under the isobaric–isothermal ensemble (NPT) after conjugate gradient energy minimization for 1000 steps and 100 ns of equilibration.,^53,54,65^ Systems were deemed equilibrated when the potential energy and volume were stabilized. Visualization and analysis were performed using VMD, ChimeraX, and web3DNA webserver.^66–68^

## 3 Results and Discussion

### 3.1 IsRNA Benchmarking Using HIV-1 DIS

Prior to the first experimental HIV-1 DIS KC or ED structures being reported, computational structural models were predicted to propose mechanisms of interconversion, estimate loop–loop interaction strengths, and provide dynamical insight.^69–71^ The HIV-1 DIS KC structures were shown to be stabilized by coaxial stacking of the two stems, and the NCp7 protein was proposed to destabilize these stacking interactions to ease the conversion to ED.^31^ Others have highlighted the importance of coaxial stacking and helical bending for RNA kissing complexes in terms of structural stability and potential molecular recognition.^4,22,23,28,46–49,72–75^ Thus, our prediction assessment is framed by this foundational work focusing on both local and global structure.

For our 60 HIV-1 DIS predictions, 36 (60%) fell below the P-value threshold of ≤0.01, indicating successful predictions. The remaining 24 predictions were considered fair, or better than chance but not below the *P*-value threshold. Thus, all predictions were better than chance, which was defined by an RMSD threshold of ≤8.71 Å compared to the respective experimental structure. Considering only the top ranked predictions from each set, the average RMSD was 3.28 Å, suggesting only small deviations from the experimental reference structures. The 36 successful predictions had an average RMSD of 3.61 Å compared to the experimental structure, with the lowest RMSD (2.73 Å) structure being the top ranked HIV-1 DIS subtype F ED prediction. The average INF score for the successful predictions was 0.84 and the average clash score was 3.98, indicating that the base pair and stacking geometries were accurately modeled compared to the experimental structure with relatively few atomic overlaps comparable to levels observed in experimental structures.^76^ The 36 successful predictions consisted of 17 ED predictions and 19 KC predictions. Of the total successful predictions, 50% (18 structures) were generated using templates that incorporated the experimental HIV-1 KC and ED structures, while the remaining 50% (18 structures) were derived from predictions excluding these templates. Taken together, these results suggest that the prediction method yields equally accurate results for KC and ED structures whether the experimental structures are known, or not.

All 60 structures (5 predictions × 6 sequences × 2 complexes) were evaluated into the ″good″ class of structures according to the IsRNA energy scoring function,^18^ indicating internal consistency within the sets of predictions. Within each set of predictions, the structures are ranked according to each structure’s energy value, such that the first structure is always relatively the best. Quantitatively, the number of successful (*P*-score ≤0.01) predictions per rank was analyzed to better understand how the accuracy of each of the five predictions differs (Figure 3).

**Figure 3:**
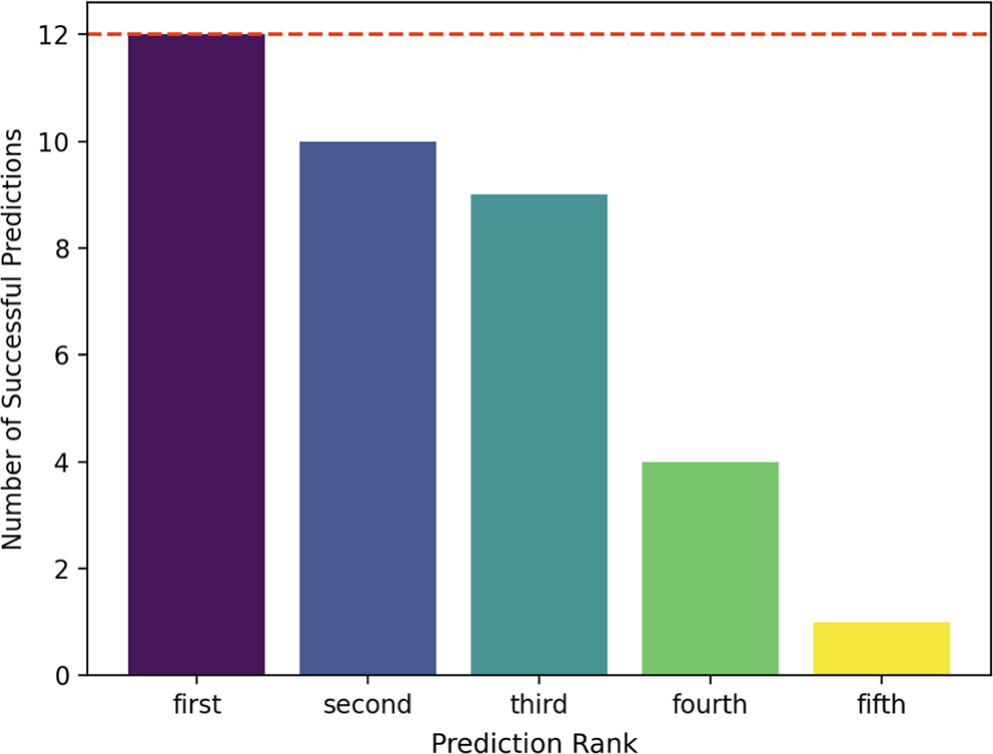
HIV-1 DIS KC or ED structural prediction success rate as measured by *P*-score over the five ranked predictions using the IsRNA model (red dotted line indicates the maximum number of predictions for each rank).

All 12 of the top-ranked, lowest-energy predictions (purple bar, Figure 3) were successful, along with ten of the second-ranked predictions and nine of the third-ranked predictions. However, accuracy dropped significantly for the fourth and fifth-ranked predictions, with only four and one successful predictions, respectively. Therefore, the top-ranked structure offers confidence, providing initial structures for molecular dynamics (MD) simulations. While our predictions achieve high scores according to two independent energy scoring functions, these values do not necessarily guarantee alignment of local structural details relative to experimental reference structures, necessitating further steps for method validation.

### 3.2 Assessing HIV-1 DIS Global vs Local Structure

Careful analysis of the regions of the predicted structures responsible for increased RMSD compared to the experimental structures is essential for understanding which types of local interactions are modeled poorly. Specifically, highly local interactions reported in the HIV-1 DIS KC and ED crystal structures are the sequence-conserved purine stacks adjacent to the kissing interaction interface that extend outside the interhelical axis.^77^ The stability of the DIS KC has been shown to depend on these base stacks, with deletion or mutation of the purines decreasing RNA dimerization, genome packaging, and viral infectivity.^71,78–81^ These stacks are not only observed in the KC structures, but also the EDs. Despite the striking global similarity of the KC or ED crystal structures between subtypes, all the reported NMR structures exhibit the purine stacking conformation within the helical axis.^24–26,28^ Structural modeling based on high resolution cryo-EM maps determined that DIS KC structures are more compact than in the crystal structures, indicating that the purines stack within the helical axis.^30^ Reported MD simulations have uncovered the ability of the stacks to adopt both “open” or closed” states, denoting the distance between the intermolecular stacks, and “bulged-in” or “bulged-out” states, denoting the inclusion or exclusion of the stacks within the helical axis.^77,82^ Thus, comparison of the predictions made in this study to experimental structures depends highly on the reference structure and the experimental method used to obtain them. When possible, comparisons to NMR structures are made, but are not to be overinterpreted since the only NMR structures available exist for the subtype B KC and ED, and subtype F KC, with a relatively high degree of heterogeneity between them which complicates direct structural comparison. Ultimately, due to the internal consistency within the many available crystal structures, main comparisons are made with crystal structures as the reference.

In all the predicted KC and ED structures, the kissing interaction-adjacent purines are stacked within the helical axis, in agreement with the NMR structures,^24–26,28^ cryo-EM maps,^30^ and solution-phase MD simulations that report the adoption of these conformations.^77,82^ Organizing the data by subtype revealed significant differences in local prediction accuracy: 65%, 35%, and 80% of predictions were successful for subtypes A, B, and F, respectively. The least successful set of predictions was the HIV-1 DIS subtype B ED structures with only two out of ten achieving success. The subtype B ED reference crystal structure for these calculations was PDB 2OIY and exhibits a “closed” conformation leading to a significant 8–13 Å difference in the distance between the C1′ atoms of the stacking purines compared to the reference structures for subtype A (462D) and subtype F (2QEK), shown to be in the “open” conformation (Figure 4A–C, top).

**Figure 4:**
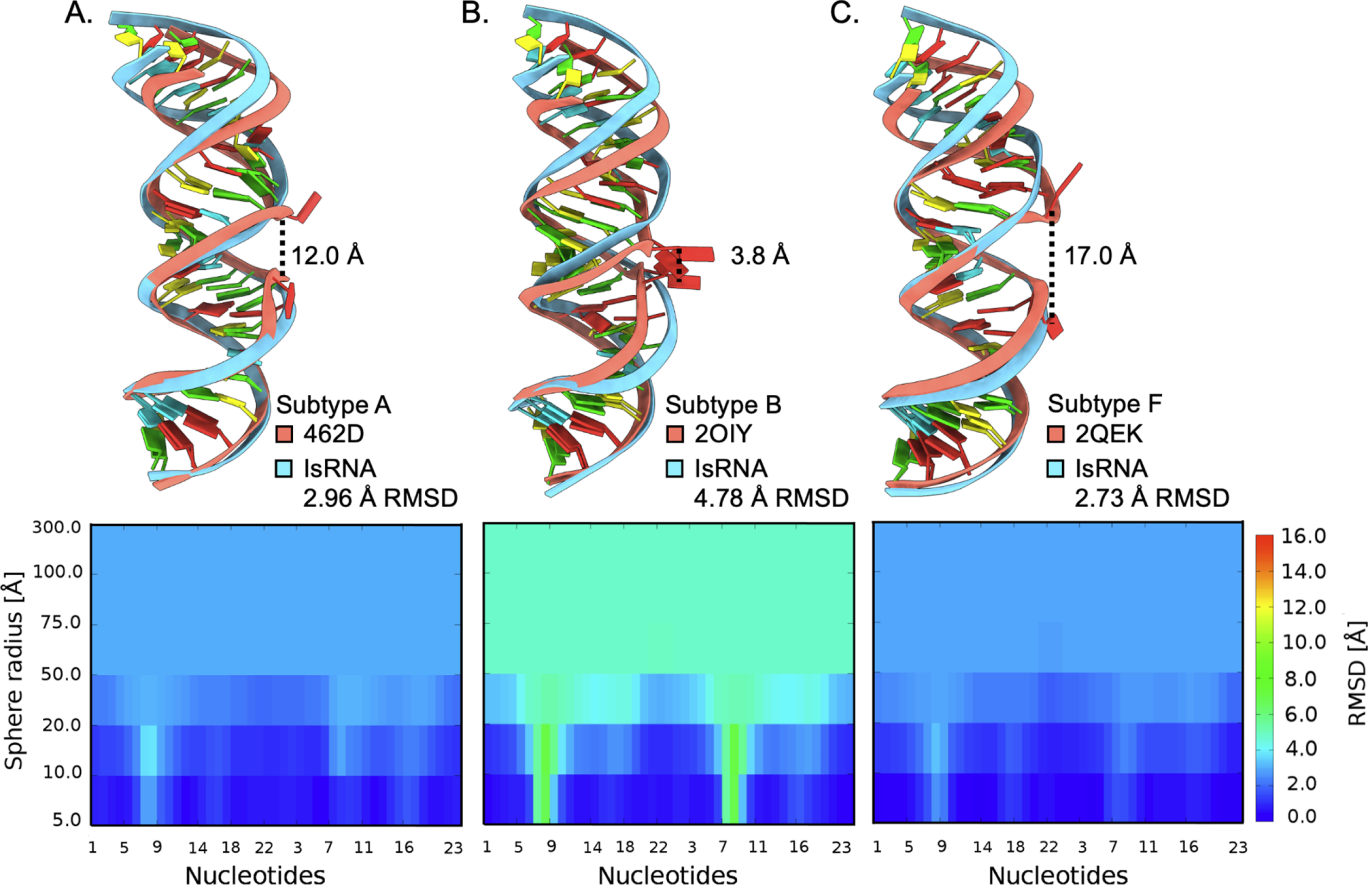
(A–C, top) Lowest RMSD ED IsRNA predictions (cyan) aligned to their respective crystal structures (salmon). Crystal structure C1′–C1′ distances of bulged-out purines indicated by dotted lines. (A–C, bottom) Local neighborhood cutoff evaluation showing contributions of local deviation to the overall global RMSD from the crystal structures centered on the bulged nucleotides (approximately nt. 7–9).

The much shorter 3.8 Å purine stack C1′–C1′ distance in subtype B results in a compression of the average helical rise (2.9 Å per base pair) compared to the other experimental ED structures (3.1–3.2 Å per base pair). Calculation of the local neighborhood cutoff evaluation measures the RMSD of all the atoms contained within a sphere centered on the C1’ atom of each nucleotide and reveals which regions of the molecule are the largest sources of RMSD when comparing calculations with spheres of varying radii (Figure 4A–C, bottom color scale). The top ranked subtype B ED prediction does not have bulged-out purines, which locally contribute to RMSD values between 6 and 8 Å according to the local-neighborhood cutoff. Thus, for eight of the subtype B ED predictions, the high structural deviation in these local regions inflates the global RMSD when calculated over all atoms, which yields *P*-score values higher than 0.01. Despite subtype A and F ED reference structures having bulged-out purine stacks, the “open” conformation results in a less compressed helical rise, and the predictions remain locally and globally similar in terms of RMSD (2–3 Å). Therefore, the difference between the reference and our predicted structures reveals the necessity of considering fine details in assessing structural prediction.

Specifically, each of the reference KC crystal structures have purine stacks in the “bulged-out, open” conformation, so the purine stacks are sources of local deviation for the predicted structures with local RMSD values of 4–5 Å while still permitting a high success rate for all subtypes (Figure 5A–C, top).

**Figure 5:**
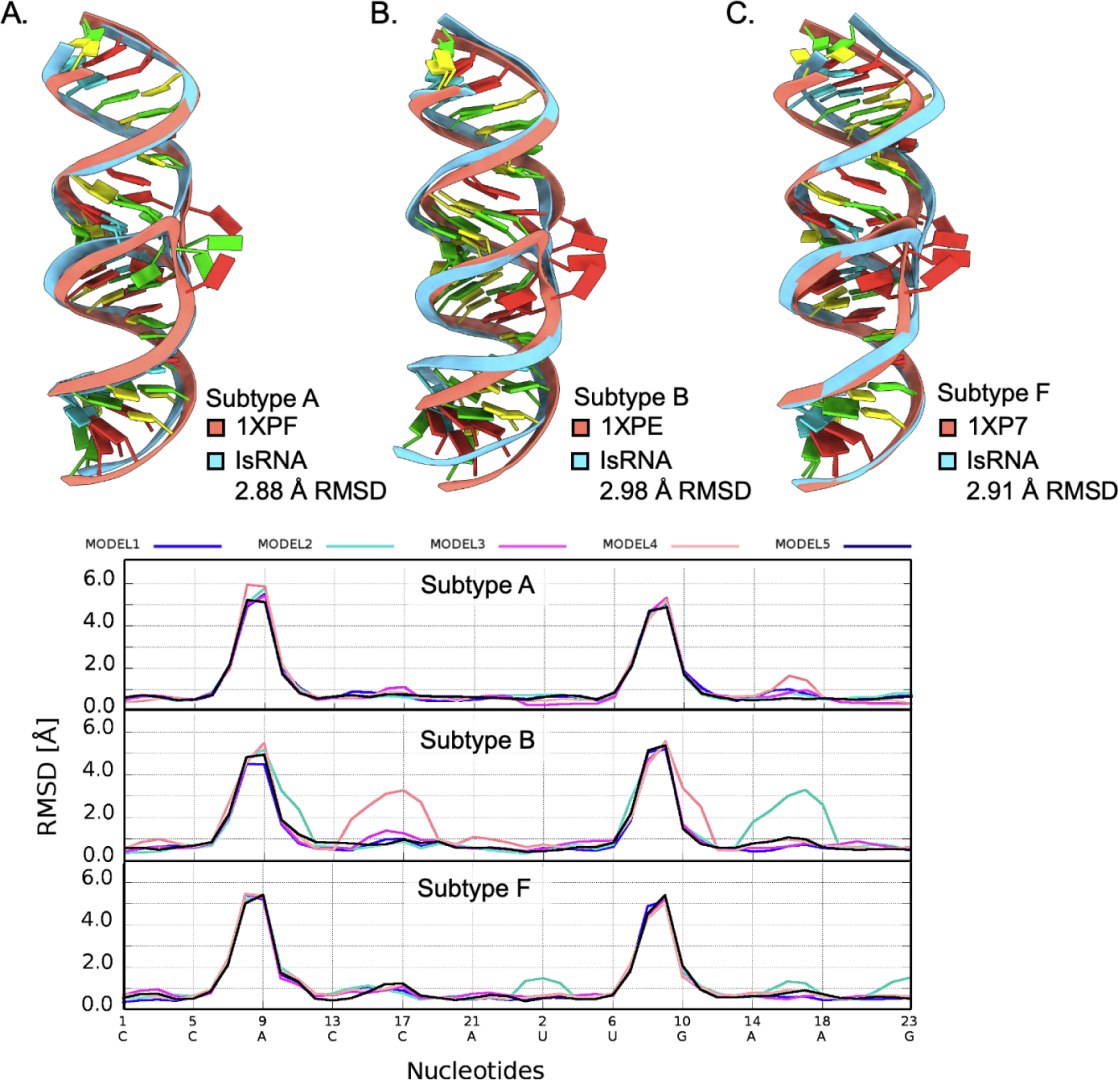
(A–C, top) Lowest RMSD KC IsRNA predicted models (cyan) aligned to their respective crystal structures (salmon). 10 Å radius local neighborhood cutoff showing contributions of local deviation to the overall global RMSD from the crystal structures centered on the bulged nucleotides (approximately nt. 7–9).

The local neighborhood cutoff with a radius of 10 Å illustrates the contributions of local deviation contained only within the bulged region (Figure 5A–C, bottom). Due to the predictions having a “bulged-in” conformation, comparison of the DIS terminal loops to the NMR structures were performed to assess agreement with the solution phase conformation. The top ranked subtype B and F KC predictions had 2.65 Å and 2.41 Å RMSD, respectively compared to 1BAU and 2D1B terminal loop nucleotides 8–15. The index of selectivity was limited to the terminal loops not only because both NMR structures differed in sequence length compared to the predictions but also in sequence identity in the stem regions (Figure 6).

**Figure 6:**
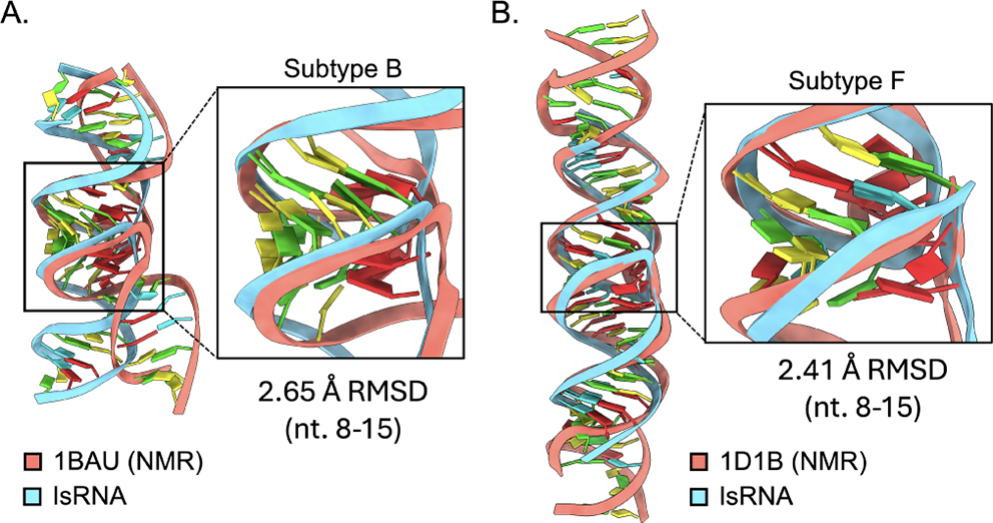
Comparison of IsRNA predicted HIV-1 DIS subtype B and F KC terminal loops with bulged-in purine stacks to NMR structures with the same conformation. Only terminal loops were compared due to stem sequence differences and length compared to NMR structures.

Despite differences in global structural alignment due to variations in stem sequence and length, the low RMSD (2.65 Å and 2.41 Å) of the terminal loop relative to the NMR structures indicates excellent agreement with solution-phase experiments and MD simulations. Furthermore, the exceptionally low RMSD values (Figure 5A–C; RMSD < 1 Å) for the remaining global fold compared to crystal structures reinforce the reliability of the structural prediction method. Comparing the top ranked predictions for both KC and ED in this work, all except for the subtype B ED had RMSD values of 2.73–2.98 Å, slightly out-performing previously reported predictions (2.9–3.3 Å), which were made without the IsRNA module of the prediction method.^31,32^ Thus, these results support the Vfold3D/LA-IsRNA approach as suitable for accurately predicting KC and ED structures of medium length, even in the absence of known experimental structures.

### 3.3 IsRNA Extension Using SARS-CoV, SARS-CoV-2, and Delta s2m

In the absence of experimental structures for the SARS-CoV, SARS-CoV-2, and Delta variant s2m dimers, predicting KC and ED structures using the validated VFold3D/LA-IsRNA pipeline will help reveal the structural and dynamical details of these viral systems of current global health interest. Of the 30 total predictions made for the s2m KC and ED structures, 27 were classified as ″good″ by the IsRNA energy scoring function, yielding a 90% success rate in the blind screening. One SARS-CoV-2 KC, one Delta KC, and one Delta ED were ″poor″ predictions, falling outside of the 0.975 × *E*_min_ relative energy threshold. Structural alignment of each of the five predictions per set (Figure 7A–C) highlights the different global folds between SARS-CoV, SARS-CoV-2, and Delta s2m KC structures.

**Figure 7:**
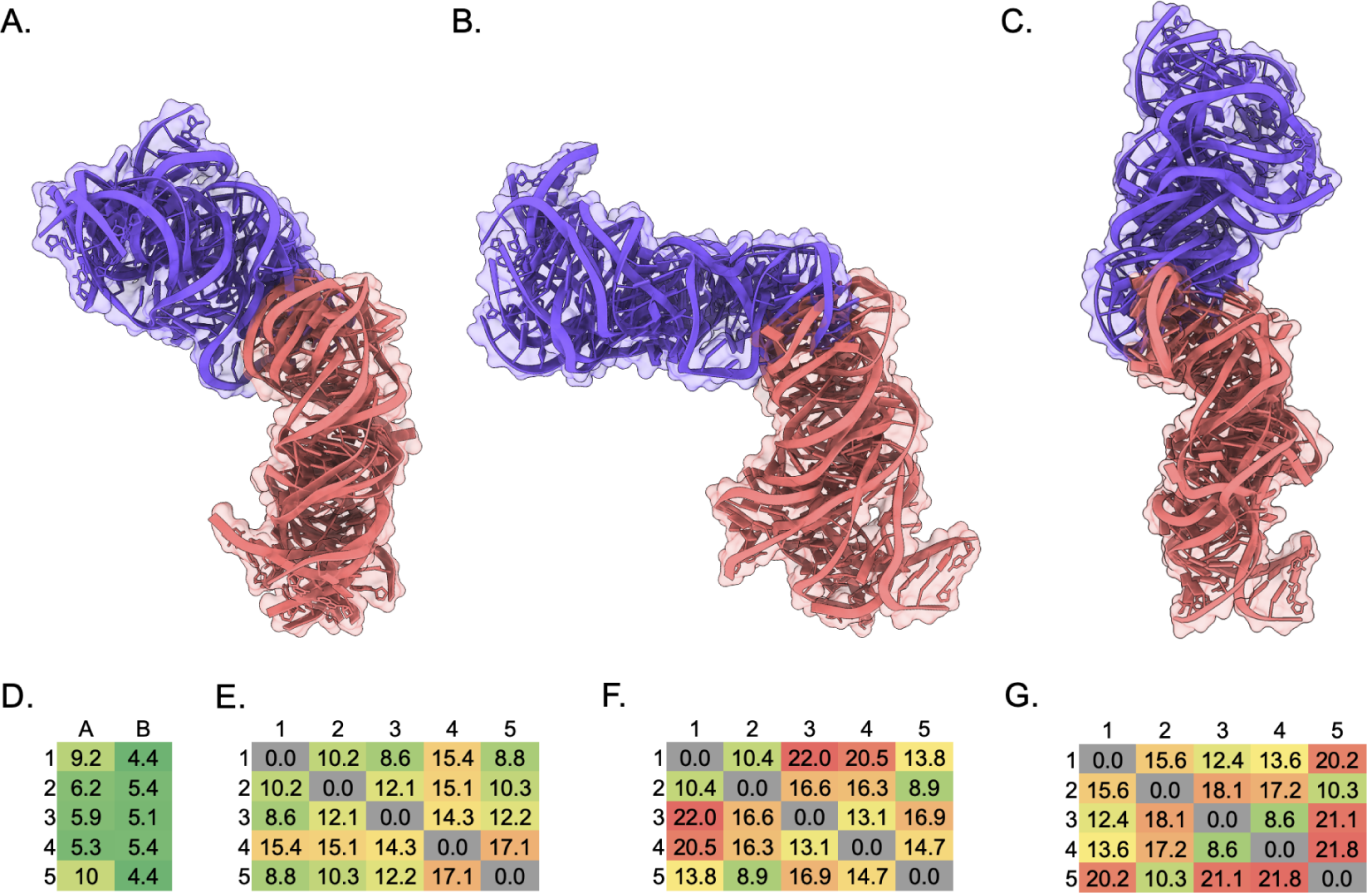
All-atom alignment of five IsRNA predicted (A) SARS-CoV s2m KC, (B) SARS-CoV-2 s2m KC, (C) Delta s2m KC. (D) RMSD in Å of each monomer A or B (excluding terminal loop nt. 17–27) in the KC models compared to the 1XJR SARS-CoV s2m crystal structure. (E–G) Pairwise all-atom RMSD matrix in Å of each predicted model to the other predictions within the set colored from lowest RMSD (green) to highest RMSD (red).

To quantitate the similarity of the monomeric hairpins within the SARS-CoV s2m KC to the 1XJR crystal structure,^8^ all heavy-atom RMSD of each monomer excluding the terminal loop nucleotides 17–27 was calculated (Figure 7D). Notably, the monomer labeled B in each of the five predictions was within 5.4 Å RMSD, indicating relative agreement for the stem structure compared to the crystal structure.

All heavy-atom pairwise RMSD was calculated for each prediction within a set of structures to assess structural variation (Figure 7E-G). The SARS-CoV s2m KC prediction showed the lowest RMSD values compared within the set of structures with a maximum value of 17.1 Å, as expected given the presence of the 1XJR s2m monomer within the template library.^8^ SARS-CoV-2 and Delta s2m KC predictions had higher variation within the respective sets (Figure 7D-G, indicated by color scale), with 22.0 and 21.8 Å RMSD as the maximum values. A moderate degree of variation is expected, since the VFold3D/LA-IsRNA pipeline utilizes coarse-grained MD simulations to sample the ensemble beyond the initial predictions. Thus, the higher degree of variation is likely due to increased dynamics within these simulations considering the bulges within the lower stems of the monomers for the SARS-CoV-2 and Delta s2m KC secondary structures, consistent with our prior observations of the s2m monomer relative entropies.^53,54^

To quantify differences in shape of the predicted KC structures, we defined a KC angle between the centers of mass of the lower stems of the monomers [(nt. 1–5, 37–41) and (nt. 42–46, 78–82)] and the palindromic nucleotides in the terminal loops [(nt. 20–23) and (nt. 61–64)] (Figure 8).

**Figure 8:**
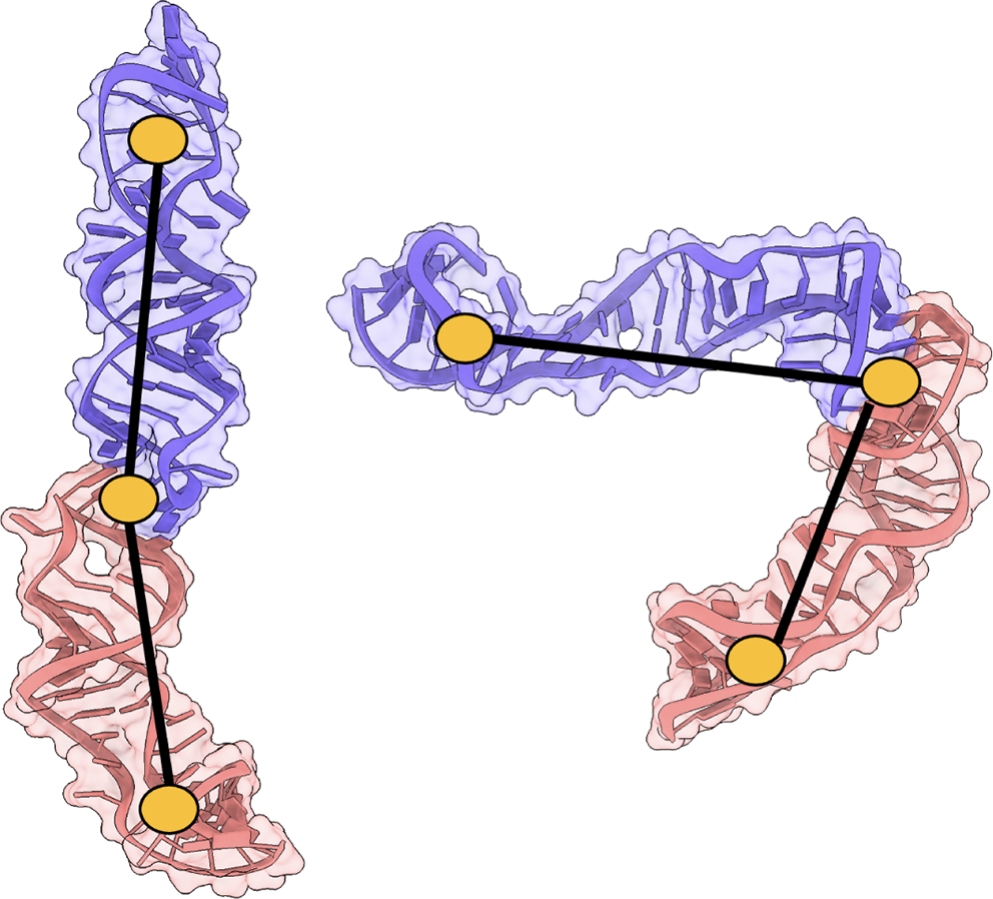
Comparison of representative KC kink angles, defined by the centers of mass of the lower stems (nt. 1–5, 37–41) and (nt. 42–46, 78–82) and the terminal loop nucleotides (nt. 20–23, 61–64), with an approximately linear KC (left) and a kinked structure (right).

The top ranked predictions had angles of 124°, 64°, and 133° with mean values over the sets (excluding structures of “poor” quality) of 126°, 105°, 142° for the SARS-CoV, SARS-CoV-2, and Delta s2m KC predictions respectively, revealing that both the top ranked and average KC bend angle for SARS-CoV-2 is significantly less obtuse compared to SARS-CoV and Delta s2m. These KC kink angle trends are in alignment with our previous observations pertaining to the monomer kink angles,^53,54^ where significant loop deformation is not observed in the predicted Delta KCs, resulting in a more linear KC structure, while both SARS-CoV and SARS-CoV-2 KCs form a kinked conformation.

In contrast, the aligned ED structures for each set show a high degree of helical stacking with less pronounced helical bending centered around palindromic sequences (Figure 9A–C), where the ED angle is defined analogously by [(nt. 1–5, 78–82) and (nt. 37–41, 42–46)] and the palindromic nucleotides in the terminal loops [(nt. 20–23) and (nt. 61–64)]. Pairwise RMSD (Figure 9D-F) again shows that the SARS-CoV s2m ED predictions have the least variation within the set of predictions with a maximum RMSD of 13.4 Å compared to 16.2 Å and 17.4 Å for SARS-CoV-2 and Delta, respectively. As seen in the aligned ED structures, much of the variation within a set is contained within the terminal 3′- and 5′-terminal ends of the monomer sequences. Despite these terminal end variations, the global bends of the top predicted structures were 156°, 127°, 127° for the SARS-CoV, SARS-CoV-2, and Delta s2m ED predictions, respectively. The mean values over the sets (excluding structures of “poor” quality) were 127°, 146°, 132° for the SARS-CoV, SARS-CoV-2, and Delta s2m ED predictions respectively, revealing less variation between the overall shapes. As a reference, an angle of 150° was calculated using this definition for the NMR structure (2D1A) of an elongated HIV-1 DIS ED of similar sequence length to the predicted s2m ED.^28^

**Figure 9:**
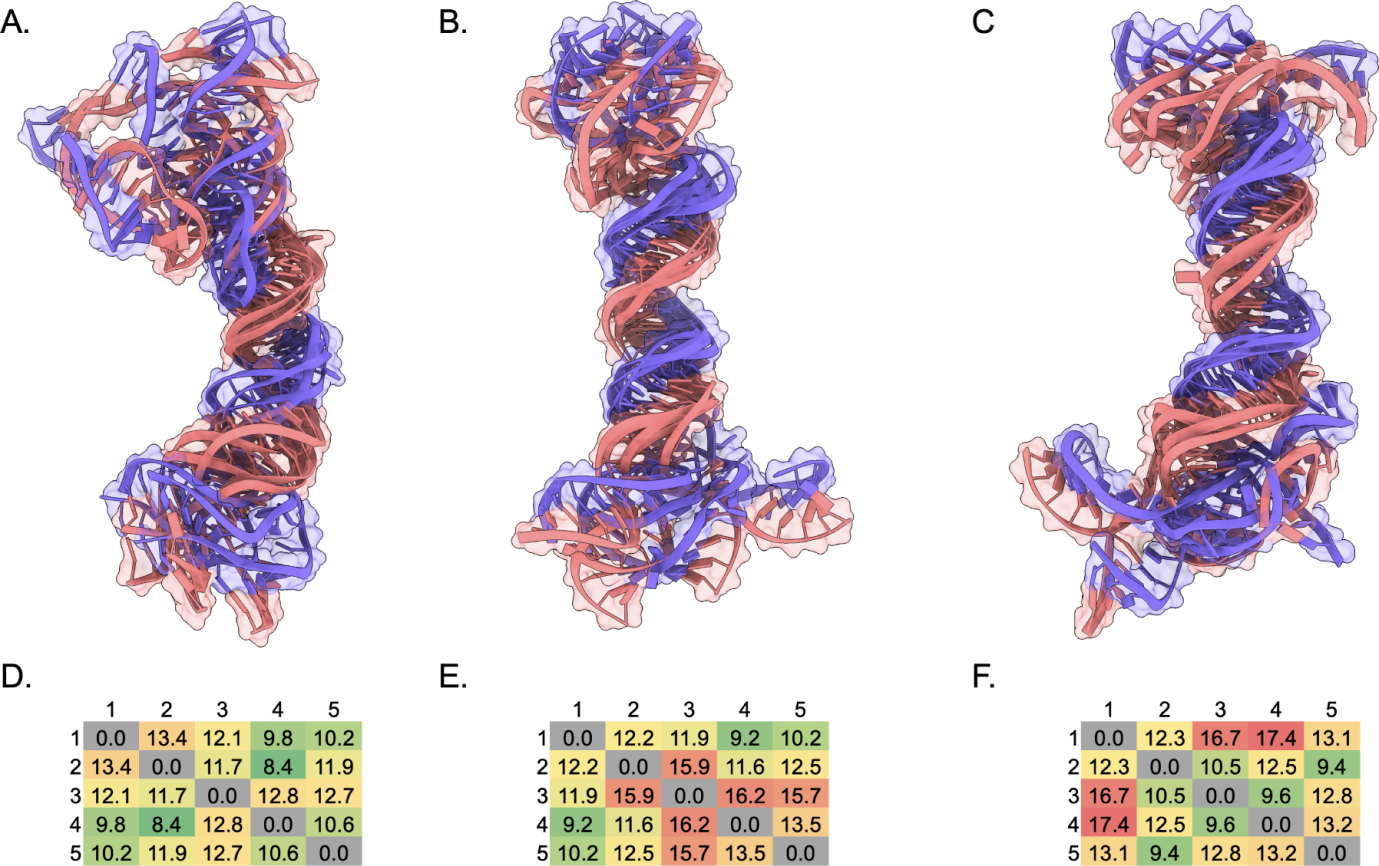
Aligned five IsRNA predicted (A) SARS-CoV s2m ED, (B) SARS-CoV-2 s2m ED, (C) Delta s2m ED. For clarity, only nt. 12–32 were used in alignment due to structural heterogeneity in the 3′- and 5′-terminal ends. (D–F) Pairwise all-atom RMSD matrix in Å of each predicted model to the other predictions within the set colored from lowest RMSD (green) to highest RMSD (red).

The shape of KC and ED structures is an important characteristic of kissing interactions because coaxially stacked helices and the linearity of the duplexes have been proposed to impact the stability of the conformations in conversion to extended duplex for several systems that form these structures.^4,22,23,28,46–49,72–75^ Our previous computational studies of the s2m monomers indicated that SARS-CoV and SARS-CoV-2 s2m have kinked shapes while the Delta s2m monomer has a linear shape due to a G to U mutation in the upper stem.^53,54^ These differences in the monomeric s2m shape were the first step in rationalizing experimental native PAGE results that suggested differences in migration of the homodimers based on shape, since they have the same molecular weight.^5,12^ To further investigate the flexibility of each KC and how the interhelical kinks observed in these structures differ, MD simulations were initiated using the top predicted structures as starting points.

### 3.4 Differences in Global Shape, Bending Dynamic, and Interhelical Kissing Interactions from Molecular Dynamics Simulations

Each of the top three ranked (lowest energy) structures were used to initialize individual 1 μs MD simulations for each of the SARS-CoV, SARS-CoV-2, and Delta s2m KC predictions resulting in a total of 3 μs per set. The first set of comparisons only considers the 1 μs MD simulations of the top ranked predictions for the highest confidence data, and a second set of comparisons involves the concatenation of all 3 μs per set, comparing a total of 9 μs of simulated time. By incorporating MD data started from the second and third ranked structure for each set, we obtain additional sampling of the potential ensemble for each KC based on different starting geometries.

Using the kink angle defined for the starting structures, the angle was measured for each frame of the KC trajectories. The angle distributions from the top ranked prediction trajectories (Figure 10A) and the concatenated top three prediction trajectories (Figure 10B) show that SARS-CoV and SARS-CoV-2 s2m KC share nearly identical average kink angles.

**Figure 10:**
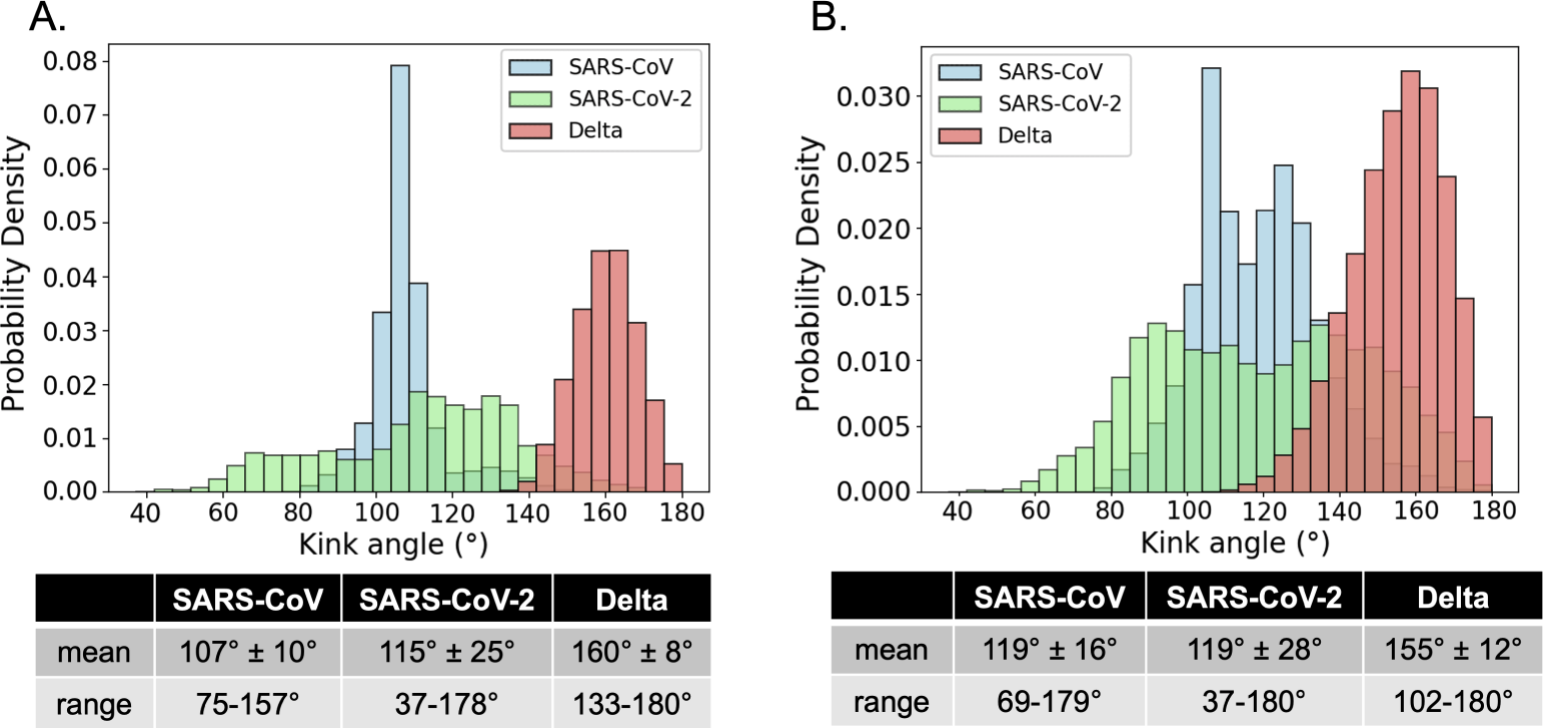
Probability density histogram of the KC kink angles measured over (A) each 1 μs MD trajectory started from the top ranked predictions (*n* = 5000 per distribution) or (B) the three concatenated 1 μs trajectories for each virus started from the top three ranked structures (*n* = 15000 per distribution).

Delta s2m KC adopts a more linear conformation of 155–160°, has smaller standard deviations from its mean of 8–12°, and samples a smaller range of angles 102–180°. This indicates a less dynamic bend motion about the interhelical axis. In both sets of comparisons, the SARS-CoV-2 s2m KC demonstrates flexibility with angle standard deviations of 25–28° about more pronounced kinked mean angles of 115–119°. The SARS-CoV KC deviates only 10–16° from its means of 107° and 119°.

As another measure of molecular shape or compactness and related dynamics that could serve as a more direct comparison to the migration of the RNA dimers within PAGE experiments, the radius of gyration from each set of trajectories was calculated (Figure 11A,B).

**Figure 11:**
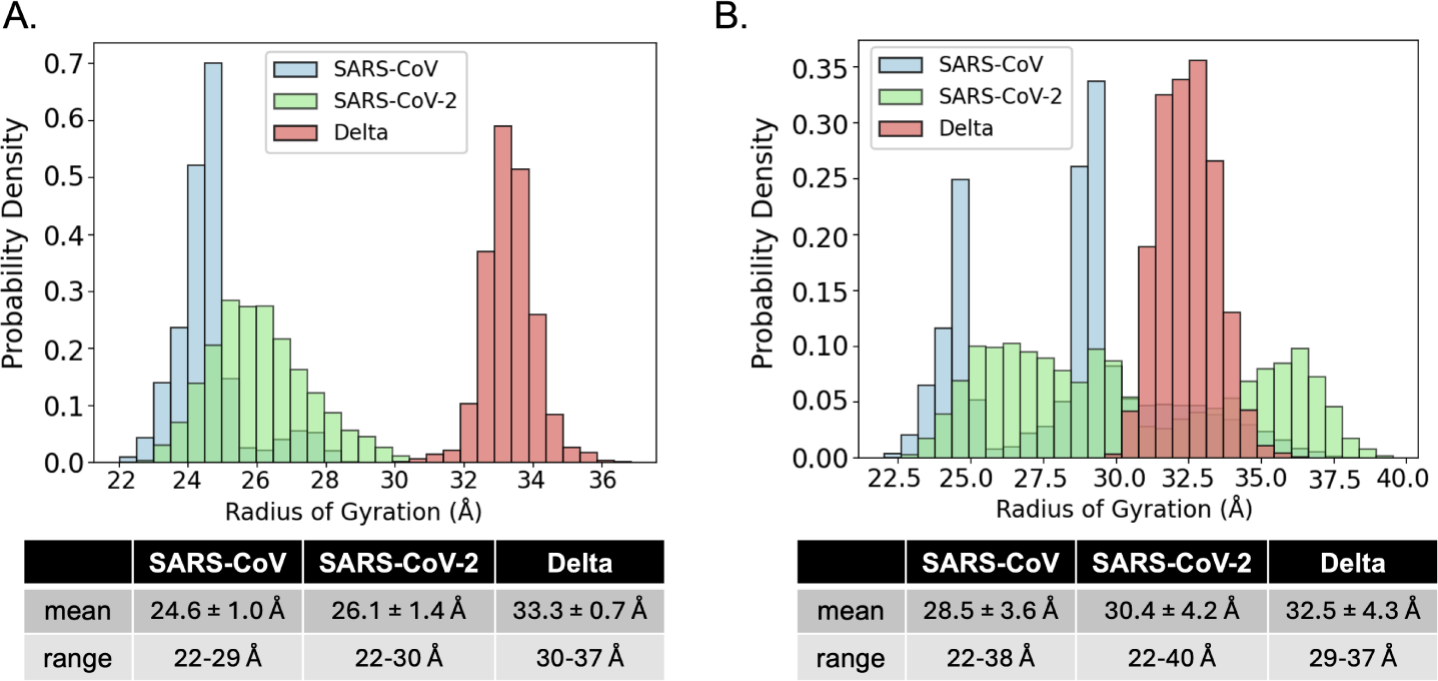
Probability density histogram of the KC radii of gyration measured over (A) each 1 μs MD trajectory started from the top ranked predictions (*n* = 5000 per distribution) or (B) the three concatenated 1 μs trajectories for each virus started from the top three ranked structures (*n* = 15000 per distribution).

A similar trend is observed in these distributions, with Delta s2m KC having larger average radii of gyration of 33.3 Å and 32.5 Å compared to those of SARS-CoV and SARS-CoV-2 s2m KC which remain below ∼30 Å. Additionally, the range of sampled radii is much smaller for Delta s2m KC indicating less deviation in its less-compacted shape compared to the other two models.

These results, while limited due to the short time scale of our simulations, suggest that SARS-CoV and SARS-CoV-2 s2m KC have similarly kinked shapes, whereas the Delta s2m KC has a less kinked shape closer to the ED. This is in agreement with predictions made about the molecular shapes of the monomers that we previously reported and also experimental native PAGE homodimerization results.^5,12^ These experiments showed that the proposed SARS-CoV-2 s2m KC migrates on the gel separately from its corresponding ED structures, suggesting differences in shape since they have identical molecular weights.^5,12^ Additionally, the Delta s2m dimerization showed only one band, suggesting that that either both the KC and ED have the same shape, or that only one of the two conformations is formed.^5^ In further support of this hypothesis, the average Delta KC angles of 155° and 160° predicted in this study from MD simulation are nearly identical to the predicted Delta s2m ED angle of 154° indicating that the two conformations would have the same overall shape. For SARS-CoV-2, the average KC angles from MD simulation of 115° or 119° are dissimilar to the predicted ED angle of 146° meaning that the two bands resolved from the native PAGE could be explained by the differences in shape predicted in this work.

To better understand how the hinge at the intermolecular interface plays a role in the global bending dynamic, the terminal loops of the lowest energy KC structures were analyzed in detail. The SARS-CoV s2m KC initial starting point had all four palindromic base pairs formed with flanking purines excluded from any stacking or base pairing interactions (Figure 12A). Unexpectedly, within the first 300 ns of MD simulation, the SARS-CoV kissing interactions through the terminal loops (nt. 17–27 and 58–68) adopted base-stacked triplet interactions at each of the palindromic base pairs (Figure 12B).

**Figure 12:**
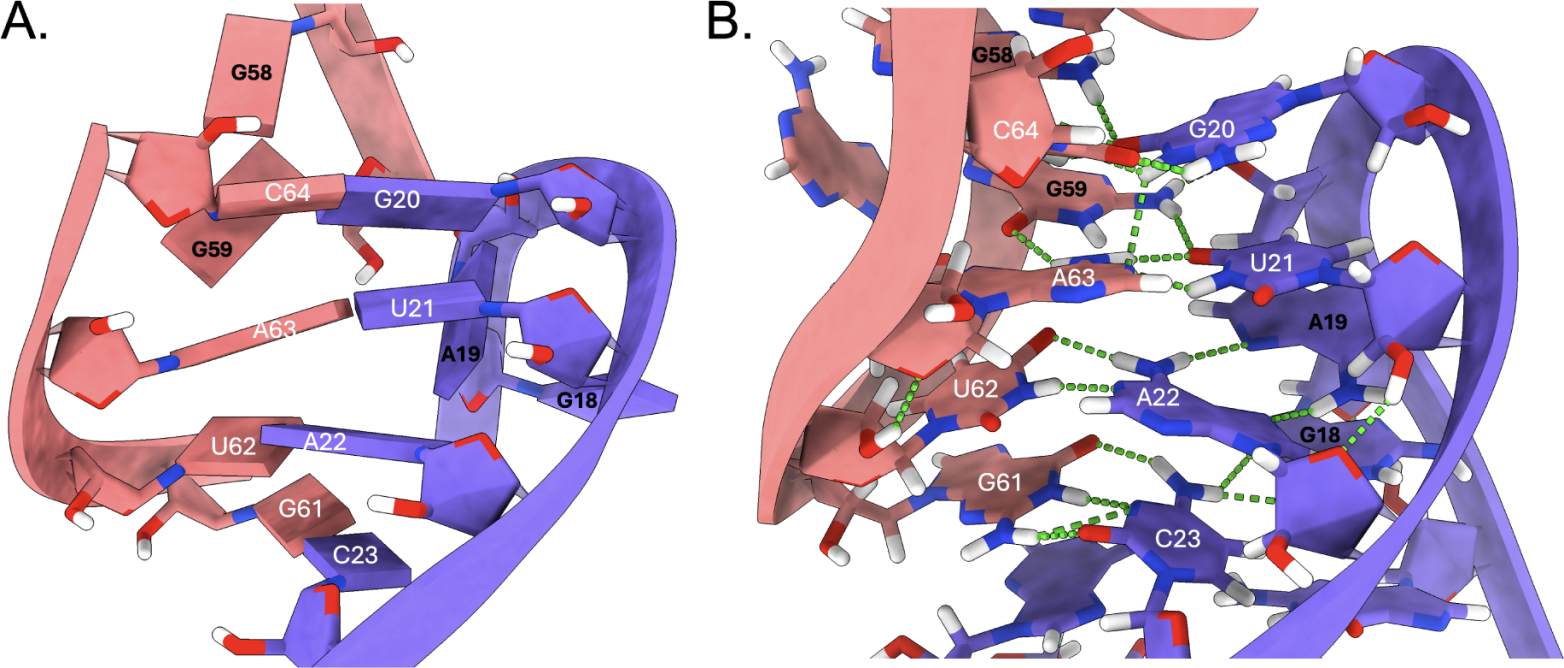
(A) Starting geometry of predicted lowest energy SARS-CoV s2m KC terminal loop for 1 μs MD simulation. (B) Base-triplet kissing interactions adopted by the SARS-CoV s2m KC involving the GUAC palindromic nucleotides (nt. 20–23, 61–64) and flanking purines (nt. G18, A19, G59, G58). Individual monomer s2m hairpins are shown in salmon and purple with hydrogen bonds shown as green dashed lines.

This complex network of hydrogen bonding and base stacking involves 12 nucleotides including both s2m GUAC palindromes and two purines flanking each respective palindrome (nt. G18, A19, G58, G59) (Figure 13).

**Figure 13:**
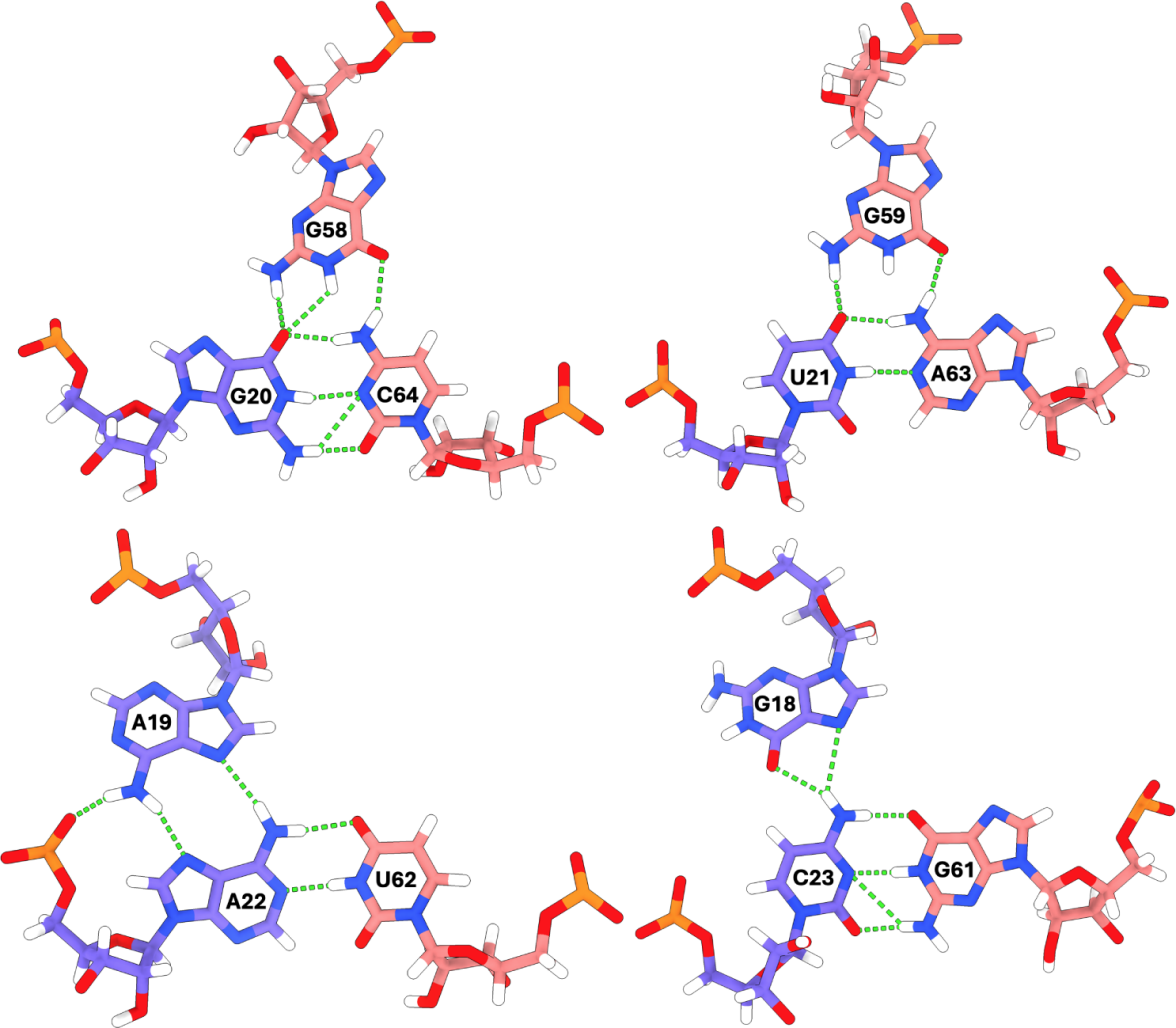
Each of the four palindromic base pairs in the SARS-CoV s2m KC is stabilized by a triplet interaction through one of the flanking purines in the terminal loop. Nucleotides are colored according to their respective monomer s2m hairpins (salmon and purple) and hydrogen bonds are shown as green dotted lines.

The involvement of flanking purines in stabilizing kissing interactions is reflective of those shown to be critical for the HIV-1 DIS KC.^4,22,23,28,46–49,72–75^ More specifically, base triplets have also been previously reported to stabilize in kissing interactions in the SLI/SLV hairpins in the *Neurospora* VS ribozyme.^83^ Thus, the base triplets observed in our MD simulations for SARS-CoV s2m KC are structurally comparable to other RNA systems and may rationalize reported experimental dimerization characteristics. In reported native PAGE experiments, the SARS-CoV s2m is shown to exist predominantly in the dimeric form showing only one migration band, indicating that the KC is favored to the monomer or ED form.^5,12^ In contrast, the SARS-CoV-2 and Delta s2m exist predominantly in the monomeric form, suggesting that any kissing interactions formed are less stable compared to SARS-CoV s2m.^5,12^ The predicted KC structures for these systems do not adopt the palindromic base triplets observed for SARS-CoV s2m, illustrating a significant relative lack of inter- and intramolecular hydrogen bonding and base stacking (Figure 14).

**Figure 14:**
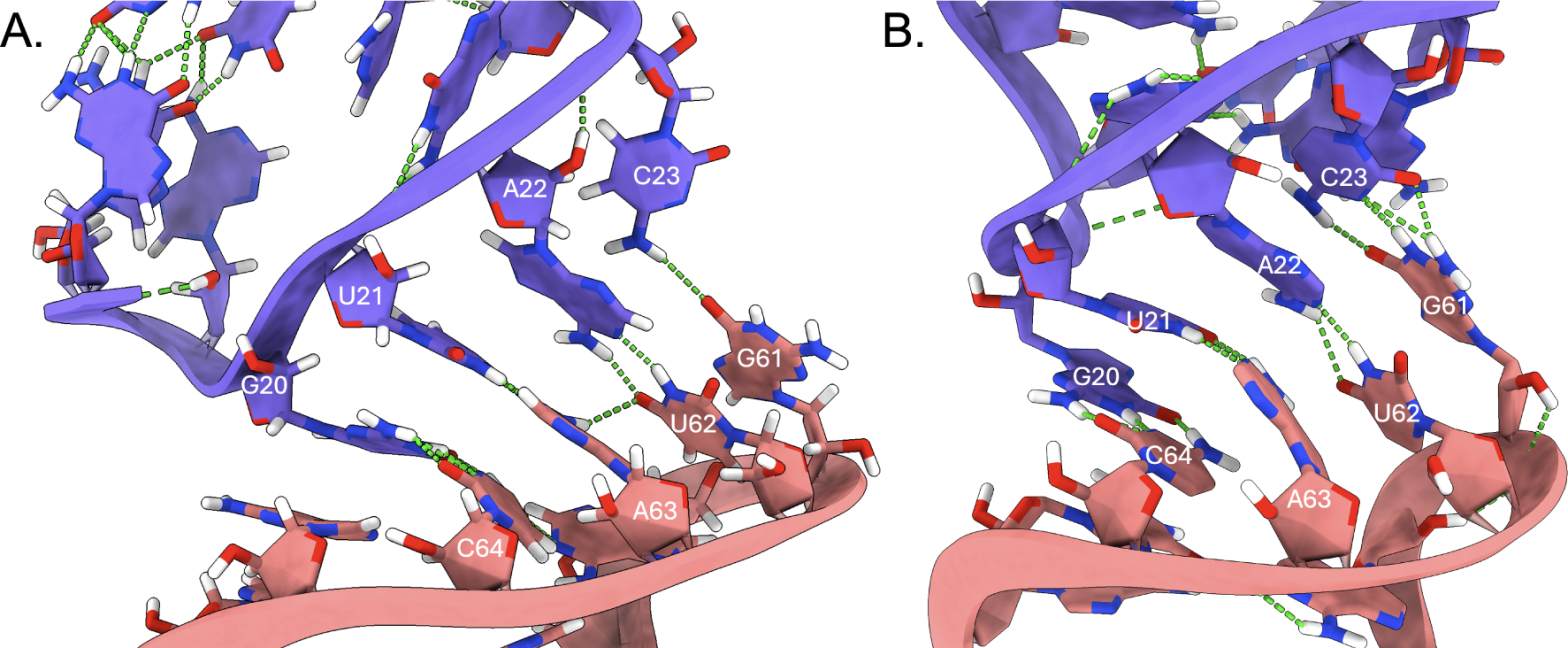
(A) SARS-CoV-2 s2m KC and (B) Delta s2m KC palindromic base pairs are not involved in base triplets as observed for SARS-CoV s2m KC. Nucleotides are colored according to their respective monomer s2m hairpins (salmon and purple) and hydrogen bonds are shown as green dotted lines.

Hydrogen bond occupancy was calculated over the MD simulations for the palindromic base pairs and triplet interactions for SARS-CoV s2m to compare relative stabilities of the intermolecular base pairs. The triplet interactions result in 20 additional stabilizing hydrogen bonds that are not observed in the SARS-CoV-2 or Delta s2m KC simulations (Table S3).

The increased dynamics and decreased palindrome hydrogen bond occupancy of SARS-CoV-2 s2m KC further supports the rationalization that the monomeric form is favored over the KC due to instability. The high base pair occupancy and low RMSD of the Delta s2m KC could suggest that the KC does persist in the homodimerization experiments, but due to having the same shape as the ED form, only one dimeric band can be resolved.

## 4 Conclusions

Our benchmarking results show that the VFold3D/LA-IsRNA pipeline reliably predicts RNA HIV-1 DIS KC and ED structures, as demonstrated by top-scoring HIV-1 DIS models achieving an average RMSD of 3.3 Å relative to crystal structures. While the crystallographic “bulged-out” purine stacks in DIS were not captured, our predicted “bulged-in” conformation aligns with reported solution-phase NMR structures, high resolution cryo-EM maps, and MD data, validating the method’s utility for modeling dynamic RNA dimer interfaces.

Building on this validation, we applied the same approach to generate the first structural models of the SARS-CoV, SARS-CoV-2, and Delta s2m KCs and EDs. Our simulations reveal distinct structural and dynamical differences across s2m variants. Our structural prediction and simulation work showed the SARS-CoV s2m KC forms a compact, kinked structure stabilized by rigidly stacked palindromic base triplets, while both SARS-CoV-2 and Delta KCs are more flexible and limited to canonical base pairing. Notably, while the SARS-CoV-2 s2m shows a conformational distinction between its kinked KC and linear ED, both Delta structures remain linear, correlating with experimental data revealing distinct complex bands for SARS-CoV-2 s2m but not for Delta s2m. These structural and dynamical features correlate with PAGE results suggesting SARS-CoV s2m favors stable dimer formation, while SARS-CoV-2 and Delta s2m exist primarily as monomers with only minor KC or ED populations. These findings validate the predictive power of the VFold3D/LA-IsRNA pipeline for RNA complexes and establish a structural basis for understanding RNA hairpin dimerization and to enable identification of targets for disrupting RNA-mediated steps of viral lifecycles.

## Supplementary Material



## Notes

Description of prediction assessment criteria calculations; HIV-1 KC or ED structures deposited in PDB; sequences and dot bracket secondary structures for benchmarking HIV-1 prediction; MD simulation palindrome hydrogen bond occupancies (PDF)
