# Supplementary material for: Structural Prediction of Coronavirus s2m Kissing Complexes and Extended Duplexes
Source: ACS Phys Chem Au. 2025 Jun 5;5(4):410–24. doi: 10.1021/acsphyschemau.5c00031 (PMC12291111; doi:10.1021/acsphyschemau.5c00031)
Supplement: Supplementary file 1 [file pg5c00031_si_001.pdf]

Structural Prediction of Coronavirus s2m Kissing Complexes and Extended Duplexes

Adam H. Kensinger, Joseph A. Makowski, Mihaela Rita Mihailescu, and Jeffrey D. Evanseck*

Department of Chemistry and Biochemistry, Duquesne University, Pittsburgh, Pennsylvania, 15282, USA

* To whom correspondence should be addressed. Tel: +14123966337; Fax: +14123965683; Email: evanseck@duq.edu

Supporting Information

Content

Supporting Methods S2

Referenced Structural Prediction Assessment Calculations Through RNAssess[1] S2

RMSD local neighborhood cutoff S2

Interaction Network Fidelity (INF) S2

Clashscore S2

P-score S3

Table S1. HIV-1 KC or ED Structures Deposited in the PDB S4

Table S2. IsRNA input HIV-1 DIS sequences and secondary structures S6

Table S3. Hydrogen bond occupancies of KC palindrome base pairs (black) and triplet interactions (gray) from MD simulations. S7

References: S8

Supporting Methods

Referenced Structural Prediction Assessment Calculations Through RNAssess[1]

RMSD local neighborhood cutoff

To assess structural agreement ranging from local to global levels between an IsRNA prediction and a reference experimental structure, RNAssess was used to obtain a neighborhood-based root mean square deviation (RMSD) metric.[1] This metric is calculated for individual nucleotides based on the spatial arrangement of surrounding atoms within a defined spherical cutoff. For each nucleotide, spheres with radii ranging from 5-300 Å are centered on the C1′ atom of the nucleotide in the reference structure. All atoms from any residue contained within each sphere are identified in both the model and reference structures. The RMSD between the prediction and reference neighborhoods is computed after alignment. The RMSD is defined as:

$$RMSD = \sqrt{\frac{1}{N}\sum_{i=1}^{N}(x_i^{\text{pred}} - x_i^{\text{ref}})^2}$$

Interaction Network Fidelity (INF)

The interaction network fidelity (INF) between an IsRNA prediction and the corresponding experimental reference was quantified using the Matthews correlation coefficient (MCC) as described by Gorodkin et al. and implemented in RNAssess.[1–3] The sets of base-pairing and base-stacking interactions, described by the Leontis-Westhof notation,[4] from the reference structure and the predicted structure were compared to calculate predicted true positives (TP), false positives (FP), and false negatives (FN). The positive predictive value (PPV), or specificity

$$\text{PPV} = \frac{|TP|}{|TP| + |FP|}$$

and sensitivity (STY) was calculated as

$$\text{STY} = \frac{|TP|}{|TP| + |FN|}$$

The MCC was then calculated as

$$\text{MCC} = \sqrt{\text{PPV} \times \text{STY}}$$

which quantifies the overall interaction fidelity between the predicted and reference structures. When the prediction reproduces all the base interactions of the reference, the MCC equals 1, indicating perfect fidelity. Conversely, if no interactions are reproduced, MCC equals 0. The interaction network fidelity (INF) between predicted and reference structures is defined as

$$\text{INF}(pred, ref) = \text{MCC}(pred, ref)$$

Clashscore

The RNAssess webserver was used to count steric clashes in the IsRNA structure predictions by calculating the clashscore, a metric derived from the MolProbity all-atom contact analysis using

the program PROBE.[1,5,6] PROBE simulates rolling a 0.5 Å diameter virtual ball around each atom's van der Waals surface to detect atomic overlaps.[5] When nonbonded, non-hydrogen donor–acceptor atoms overlap by more than 0.4 Å, the contact is flagged as a serious clash. The clashscore is then defined as the number of such clashes per 1000 atoms in the structure. This provides a quantitative measure of the physical plausibility of predicted RNA 3D models.

P-score

To evaluate the statistical significance of RNA structure predictions in this work, we calculated P-score developed by Hajdin et al. (2010)[7] using the RNAssess webserver implementation.[1] P-score quantifies the likelihood that a predicted RNA tertiary structure is significantly more accurate than a random RNA fold, given the chain length and the observed root-mean-square deviation (RMSD) from a reference structure.

For this assessment metric calculation, the expected RMSD for structures of a given nucleotide sequence length *N* is described by:

$$\langle \mathrm{RMSD} \rangle = \mathrm{a} \cdot \mathrm{N}^{0.41} - \mathrm{b}$$

where constants $a = 6.4$ and $b = 12.7$ are values empirically derived by Hajdin et al. based on replica exchange MD simulations of RNA structures with enforced secondary structure constraints. The exponent 0.41 is based on the scaling behavior of RNA radius of gyration with chain length, which Hajdin et al. found to be intermediate between that of compact spheres and self-avoiding polymers.[7]

To quantify how far the calculated RMSD between a given IsRNA prediction and its experimental reference structure deviates from the expected average ⟨RMSD⟩ for random RNA-like structures of the same length, the Z-score is calculated as

$$Z = \frac{m - \langle \mathrm{RMSD} \rangle}{s_m}$$

where *m* is the calculated RMSD between the predicted and experimental structure and $s_m = 1.8$ Å is the standard deviation determined empirically by Hajdin et al., who observed that the RMSD distributions for RNA ensembles of various lengths were approximately Gaussian and exhibited length-independent variance.[7] The deviation is expressed in terms of the number of standard deviations away from the mean RMSD. This standardization allows for the comparison of structure prediction significance across different RNA lengths by mapping RMSD values onto a common statistical scale. Once the Z-score is calculated, it can be converted into a probability (P-score) using the error function,

$$P = 0.5 \cdot \left[1 + erf\left(\frac{Z}{\sqrt{2}}\right)\right]$$

giving a measure of how likely the predicted structure was generated by chance. Thus, p-score allows for quantitative evaluation of whether a given structure prediction is significantly more accurate than expected by chance.[7]

Table S1. HIV-1 KC or ED Structures Deposited in the PDB.*[8–18]

	PDB	Element	Structure	Subtype	Method
1	1WVD	DIS	ED	A	Xray
2	1XPF	DIS	KC	A	Xray
3	2OIJ	DIS	ED	B	Xray
4	1XPE	DIS	KC	B	Xray
5	2F4X	DIS	KC	B	NMR
6	1JU1	DIS	ED	B	NMR
7	2QEK	DIS	ED	F	Xray
8	3C44	DIS	ED	F	Xray
9	1Y3O	DIS	KC	F	Xray
10	2D1A	DIS	ED	B	NMR
11	2D18	DIS	ED	B	NMR
12	2GM0	DIS	ED	B	NMR
13	2D1B	DIS	KC	F	NMR
14	2D19	DIS	KC	F	NMR
15	1BAU	DIS	KC	B	NMR
16	1KIS	TAR	KC	-	NMR
17	2PN9	TAR	KC	-	NMR
18	2OOM	TAR	KC	-	NMR
19	2JLT	TAR	KC	-	Xray
20	2RN1	TAR	KC	-	NMR
21	1O3Z	DIS	ED	A	Xray
22	1Y6S	DIS	ED	A	Xray
23	1Y6T	DIS	ED	A	Xray
24	1Y73	DIS	ED	A	Xray
25	1Y95	DIS	ED	A	Xray
26	1Y99	DIS	ED	A	Xray
27	462D	DIS	ED	A	Xray
28	1K9W	DIS	KC	A	Xray
29	1NLC	DIS	KC	A	Xray
30	2B8S	DIS	KC	A	Xray
31	1Y90	DIS	KC	A	Xray
32	6BG9	DIS	ED	B	NMR/EM
33	2OJ0	DIS	ED	B	Xray
34	1JU1	DIS	ED	B	NMR
35	3FAR	DIS	ED	B	Xray
36	2B8R	DIS	KC	B	Xray
37	3DVV	DIS	ED	F	Xray

38	1ZCI	DIS	KC	F	Xray
39	1Y3S	DIS	KC	F	Xray
40	1YXP	DIS	KC	F	Xray
41	1XP7	DIS	KC	F	Xray
42	2FCZ	DIS	KC	F	Xray
43	2FCX	DIS	KC	F	Xray
44	2FCY	DIS	KC	F	Xray
45	2FD0	DIS	KC	F	Xray
46	2D17	DIS	stem-bulge-stem	-	NMR

* The first 20 PDBs listed are those included in the IsRNA template library.

Table S2. IsRNA input HIV-1 DIS sequences and secondary structures.

Structure	Subtype	DIS sequence/dot bracket
KC (1XPF)	A	CUUGCUGAGGUGCACACAGCAAG-CUUGCUGAGGUGCACACAGCAAG<br>(((((((..[[[[[[.)))))))-(((((((..]]]]]].)))))))
ED (462D)	A	CUUGCUGAGGUGCACACAGCAAG-CUUGCUGAGGUGCACACAGCAAG<br>(((((((..((((((.(((((((-)))))))..)))))).)))))))
KC (1XPE)	B	CUUGCUGAAGCGCGCACGGCAAG-CUUGCUGAAGCGCGCACGGCAAG<br>(((((((..[[[[[[.)))))))-(((((((..]]]]]].)))))))
ED (2OIY)	B	CUUGCUGAAGCGCGCACGGCAAG-CUUGCUGAAGCGCGCACGGCAAG<br>(((((((..((((((.(((((((-)))))))..)))))).)))))))
KC (1XP7)	F	CUUGCUGAAGUGCACACAGCAAG-CUUGCUGAAGUGCACACAGCAAG<br>(((((((..[[[[[[.)))))))-(((((((..]]]]]].)))))))
ED (2QEK)	F	CUUGCUGAAGUGCACACAGCAAG-CUUGCUGAAGUGCACACAGCAAG<br>(((((((..((((((.(((((((-)))))))..)))))).)))))))

Table S3. Hydrogen bond occupancies of KC palindrome base pairs (black) and triplet interactions (gray) from MD simulations.

			SARS-CoV	SARS-CoV-2	Delta
**Base Pair**	**donor**	**acceptor**	**occupancy**	**occupancy**	**occupancy**
G20-C64	G20-N1	C64-N3	**70%**	**81%**	**92%**
	G20-N2	C64-O2	**75%**	**82%**	**91%**
	C64-N4	G20-O6	**66%**	**81%**	**90%**
G58	G58-N1	G20-O6	57%		
	C64-N4	G58-O6	33%		
	G58-N2	G20-O6	30%		
	G58-N2	G20-N7	4%		
	C64-N4	G20-N1	3%		
	G58-N1	C64-N4	1%		
U21-A63	U21-N3	A63-N1	**81%**	**80%**	**87%**
	A63-N6	U21-O4	**76%**	**70%**	**76%**
G59	G59-N2	U21-O4	54%		
	A63-N6	G59-O6	43%		
	A63-N6	G59-N2	23%		
	G59-N2	U62-O4	8%		
	G59-N1	A63-N6	7%		
	G59-N2	A63-N7	6%		
	G59-N1	A63-N7	3%		
A22-U62	U62-N3	A22-N1	**84%**	**65%**	**80%**
	A22-N6	U62-O4	**78%**	**37%**	**60%**
A19	A22-N6	A19-N7	81%		
	A19-N6	A22-OP2	79%		
	A19-N6	A22-N7	62%		
	G59-N2	A19-O2'	56%		
	A19-O2'	G20-N7	6%		
C23-G61	G61-N1	C23-N3	**82%**	**62%**	**91%**
	G61-N2	C23-O2	**90%**	**62%**	**87%**
	C23-N4	G61-O6	**71%**	**59%**	**87%**
G18	C23-N4	G18-O6	45%		
	C23-N4	G18-N7	11%		

References:

(1) Lukasiak, P.; Antczak, M.; Ratajczak, T.; Szachniuk, M.; Popenda, M.; Adamiak, R. W.; Blazewicz, J. RNAssess—a Web Server for Quality Assessment of RNA 3D Structures. *Nucleic Acids Res* **2015**, *43* (Web Server issue), W502. https://doi.org/10.1093/NAR/GKV557.

(2) Gorodkin, J.; Stricklin, S. L.; Stormo, G. D. Discovering Common Stem–Loop Motifs in Unaligned RNA Sequences. *Nucleic Acids Res* **2001**, *29* (10), 2135. https://doi.org/10.1093/NAR/29.10.2135.

(3) Parisien, M.; Cruz, J. A.; Westhof, É.; Major, F. New Metrics for Comparing and Assessing Discrepancies between RNA 3D Structures and Models. *RNA* **2009**, *15* (10), 1875–1885. https://doi.org/10.1261/RNA.1700409.

(4) Leontis, N. B.; Stombaugh, J.; Westhof, E. The Non-Watson–Crick Base Pairs and Their Associated Isostericity Matrices. *Nucleic Acids Res* **2002**, *30* (16), 3497–3531. https://doi.org/10.1093/NAR/GKF481.

(5) Word, J. M.; Lovell, S. C.; Labean, T. H.; Taylor, H. C.; Zalis, M. E.; Presley, B. K.; Richardson, J. S.; Richardson, D. C. Visualizing and Quantifying Molecular Goodness-of-Fit: Small-Probe Contact Dots with Explicit Hydrogen Atoms. *J Mol Biol* **1999**, *285* (4), 1711–1733. https://doi.org/10.1006/JMBI.1998.2400.

(6) Chen, V. B.; Arendall, W. B.; Headd, J. J.; Keedy, D. A.; Immormino, R. M.; Kapral, G. J.; Murray, L. W.; Richardson, J. S.; Richardson, D. C. MolProbity: All-Atom Structure Validation for Macromolecular Crystallography. *urn:issn:0907-4449* **2009**, *66* (1), 12–21. https://doi.org/10.1107/S0907444909042073.

(7) Hajdin, C. E.; Ding, F.; Dokholyan, N. V.; Weeks, K. M. On the Significance of an RNA Tertiary Structure Prediction. *RNA* **2010**, *16* (7), 1340–1349. https://doi.org/10.1261/RNA.1837410.

(8) Ennifar, E.; Walter, P.; Dumas, P. A Crystallographic Study of the Binding of 13 Metal Ions to Two Related RNA Duplexes. *Nucleic Acids Res* **2003**, *31* (10), 2671–2682. https://doi.org/10.1093/NAR/GKG350.

(9) Ennifar, E.; Yusupov, M.; Walter, P.; Marquet, R.; Ehresmann, B.; Ehresmann, C.; Dumas, P. The Crystal Structure of the Dimerization Initiation Site of Genomic HIV-1 RNA Reveals an Extended Duplex with Two Adenine Bulges. *Structure* **1999**, *7* (11), 1439–1449. https://doi.org/10.1016/S0969-2126(00)80033-7.

(10) Ennifar, E.; Dumas, P. Polymorphism of Bulged-out Residues in HIV-1 RNA DIS Kissing Complex and Structure Comparison with Solution Studies. *J Mol Biol* **2006**, *356* (3), 771–782. https://doi.org/10.1016/J.JMB.2005.12.022.

(11) Ennifar, E.; Walter, P.; Ehresmann, B.; Ehresmann, C.; Dumas, P. Crystal Structures of Coaxially Stacked Kissing Complexes of the HIV-1 RNA Dimerization Initiation Site. *Nature Structural Biology 2001 8:12* **2001**, *8* (12), 1064–1068. https://doi.org/10.1038/nsb727.

(12) Zhang, K.; Keane, S. C.; Su, Z.; Irobalieva, R. N.; Chen, M.; Van, V.; Sciandra, C. A.; Marchant, J.; Heng, X.; Schmid, M. F.; Case, D. A.; Ludtke, S. J.; Summers, M. F.; Chiu, W. Structure of the 30 KDa HIV-1 RNA Dimerization Signal by a Hybrid Cryo-EM, NMR, and Molecular Dynamics Approach. *Structure* **2018**, *26* (3), 490-498.e3. https://doi.org/10.1016/J.STR.2018.01.001.

(13) Girard, F.; Barbault, F.; Gouyette, C.; Huynh-Dinh, T.; Paoletti, J.; Lancelot, G. Dimer Initiation Sequence of HIV-1Lai Genomic RNA: NMR Solution Structure of the Extended Duplex. *J Biomol Struct Dyn* **1999**, *16* (6), 1145–1157. https://doi.org/10.1080/07391102.1999.10508323.

(14) Kieken, F.; Paquet, F.; Brulé, F.; Paoletti, J.; Lancelot, G. A New NMR Solution Structure of the SL1 HIV-1Lai Loop–Loop Dimer. *Nucleic Acids Res* **2006**, *34* (1), 343. https://doi.org/10.1093/NAR/GKJ427.
(15) Freisz, S.; Lang, K.; Micura, R.; Dumas, P.; Ennifar, E. Binding of Aminoglycoside Antibiotics to the Duplex Form of the HIV-1 Genomic RNA Dimerization Initiation Site. *Angewandte Chemie International Edition* **2008**, *47* (22), 4110–4113. https://doi.org/10.1002/ANIE.200800726.
(16) Olieric, V.; Rieder, U.; Lang, K.; Serganov, A.; Schulze-Briese, C.; Micura, R.; Dumas, P.; Ennifar, E. A Fast Selenium Derivatization Strategy for Crystallization and Phasing of RNA Structures. *RNA* **2009**, *15* (4), 707. https://doi.org/10.1261/RNA.1499309.
(17) Ennifar, E.; Paillart, J. C.; Bodlenner, A.; Walter, P.; Weibel, J. M.; Aubertin, A. M.; Pale, P.; Dumas, P.; Marquet, R. Targeting the Dimerization Initiation Site of HIV-1 RNA with Aminoglycosides: From Crystal to Cell. *Nucleic Acids Res* **2006**, *34* (8), 2328–2339. https://doi.org/10.1093/NAR/GKL317.
(18) Baba, S.; Takahashi, K. I.; Noguchi, S.; Takaku, H.; Koyanagi, Y.; Yamamoto, N.; Kawai, G. Solution RNA Structures of the HIV-1 Dimerization Initiation Site in the Kissing-Loop and Extended-Duplex Dimers. *The Journal of Biochemistry* **2005**, *138* (5), 583–592. https://doi.org/10.1093/JB/MVI158.
